# Responses to environmental variability by herbivorous insects and their natural enemies within a bioenergy crop, *Miscanthus* x *giganteus*

**DOI:** 10.1371/journal.pone.0246855

**Published:** 2021-02-16

**Authors:** Alisa W. Coffin, Dawn M. Olson, Lynne Seymour, David D. Bosch, Jason M. Schmidt, Timothy C. Strickland

**Affiliations:** 1 USDA-ARS, Southeast Watershed Research Laboratory, Tifton, Georgia, United States of America; 2 Department of Statistics, University of Georgia, Athens, Georgia, United States of America; 3 Department of Entomology, University of Georgia, Tifton, Georgia, United States of America; University of Saskatchewan College of Agriculture and Bioresources, CANADA

## Abstract

Precision agriculture (PA) is the application of management decisions based on identifying, quantifying, and responding to space-time variability. However, knowledge of crop pest responses to within-field environmental variability, and the spatial distribution of their natural enemies, is limited. Quantitative methods providing insights on how pest-predator relationships vary within fields are potentially important tools. In this study, phloem feeders and their natural enemies, were observed over two years across 81 locations within a field of the perennial feedstock grass in Georgia, USA. Geographically weighted regression (GWR) was used to spatially correlate their abundance with environmental factors. Variables included distance to forest edge, Normalized Difference of Vegetation Index (NDVI), slope, aspect, elevation, soil particle size distribution, and weather values. GWR methods were compared with generalized linear regression methods that do not account for spatial information. Non-spatial models indicated positive relationships between phloem-feeder abundance and wind speed, but negative relationships between elevation, proportions of silt and sand, and NDVI. With data partitioned into three seasonal groups, terrain and soil variables remained significant, and natural enemies and spiders became relevant. Results from GWR indicated that magnitudes and directions of responses varied within the field, and that relationships differed among seasons. Strong negative relationships between response and explanatory factors occurred: with NDVI during mid-season; with percent silt, during mid-, and late seasons; and with spider abundance during early and late seasons. In GWR models, slope, elevation, and aspect were mostly positive indicating further that associations with elevation depended on whether models incorporated spatial information or not. By using spatially explicit models, the analysis provided a complex, nuanced understanding of within-field relationships between phloem feeders and environmental covariates. This approach provides an opportunity to learn about the variability within agricultural fields and, with further analysis, has potential to inform and improve PA and habitat management decisions.

## Introduction

Agricultural field characteristics of terrain, soils, and microclimate influence outcomes such as crop yield but can be highly variable. Furthermore, while terrain and soil conditions change very slowly, weather changes quickly over the course of a season and likely influences plant and associated organisms (e.g. insects, pathogens). In short, ecological relationships within agricultural fields are in constant flux, varying over space and changing in time. In agricultural production systems, the use of insecticides remains prevalent despite the negative effects they have on non-target organisms in the environment [1–4]. Reducing reliance on broad spectrum pesticides in agriculture could occur with information on the spatial and temporal (location in field and seasonality) distribution of pests and their associations with environmental variability (i.e. plant quality, soil quality, topography) [5–16]. Spatial and temporal information on insect communities and environmental conditions could be used to better target site-specific application of water, nutrients and pest management chemistries [17] and implement within-field and field edge habitat management practices to enhance pollination and conservation biological control [18].

Precision agriculture (PA) in its broadest sense is the application of management decisions in space and time based on identifying, quantifying, and responding to variability among and within agricultural fields. The degree of precision achieved increases with the adoption of technologies such as tractor mounted or drone-based sensors [19] that provide increasingly precise data. PA can result in the more appropriate use of inputs that benefit the profitability of the enterprise and natural resource management. However, knowledge of crop pests and natural enemy variability is often generalized over entire fields, which makes it challenging to implement PA effectively for precise pest management strategies. Therefore, quantitative methods that reveal how pest/predator relationships vary in space, even within a field, are essential for advancing PA.

Such timely information can help identify zones containing localized pest outbreaks that could be mitigated before spread, thus lowering insecticide use and potentially increasing overall production. Phloem feeding insects such as aphids (Hemiptera: Aphidae), thrips (Thysanoptera: Thripidae) and whiteflies (Hemiptera; Aleyrodidae) are highly polyphagous and serious pests of numerous crop species. As sap feeders, they also vector many species of plant pathogens [20–23]. Phloem feeders are preyed upon by several important generalist insect predator and spider species that can exert significant suppression of these pests, especially when they are part of a pest management strategy [24]. Documenting the association of herbivorous insects and their natural enemies in crop fields is critical to predicting influences on neighboring crop production systems.

Production systems dedicated to bioenergy feedstocks, such as perennial grasses, have increased substantially over the last decade in response to calls for the development of a “bioeconomy” [25]. Global increases in the production of biofuel feedstock crops have prompted numerous landscape assessments in Europe and the Midwestern USA of their potential direct and indirect effects on arthropods and ecosystem function in agroecosystems [26–31]. The southeastern USA has been recognized as a highly suitable area for biofuel feedstock production [32], and several studies document arthropods associated with various biofuel crops in the southeast USA [33–38]. However, while identifying pest problems in biofuel crops, these studies do not consider the spatially explicit nature of the factors driving pest responses, or the fact that there may be spatial and temporal autocorrelations among sampled sites. Therefore, incorporating the shifting influences of environmental drivers on insect populations over space and time should provide a better, more predictable framework for applying PA to insect management.

Geographically weighted regression (GWR) is a statistical method used to account for spatial non-stationarity present in variable environments [39]. In contrast to common linear models, GWR models are equipped to estimate the magnitude and direction of explanatory factors that vary over space by weighting the effects of environmental variability by their locations within field. To date GWR has successfully predicted outcomes of heterogeneity in topography on soil contaminants [40], relationships between soil texture and organic carbon [41], and the location of insect pests in relation to plant health [42]. Therefore, GWR has the potential to assist in the quest for high-resolution within-field predictive modeling of crop-environment relationships, laying the foundation for improved PA programs [36].

In this study, we sampled canopy-dwelling and ground-dwelling insects and spiders. We determined their spatial distribution across 81 locations within a field of a perennial bioenergy feedstock grass. Our objective was to investigate insect natural enemy and spider abundance, along with environmental variables, as factors that shape within field spatial distribution of herbivorous pests, specifically phloem feeders, over the growing season within this relatively new crop. To do so, we compared outcomes of non-spatial linear regression methods with GWR models, to ascertain how precise, spatially explicit models could better predict pest-predator-environment dynamics.

## Materials and methods

### Field description

**Fig 1:**
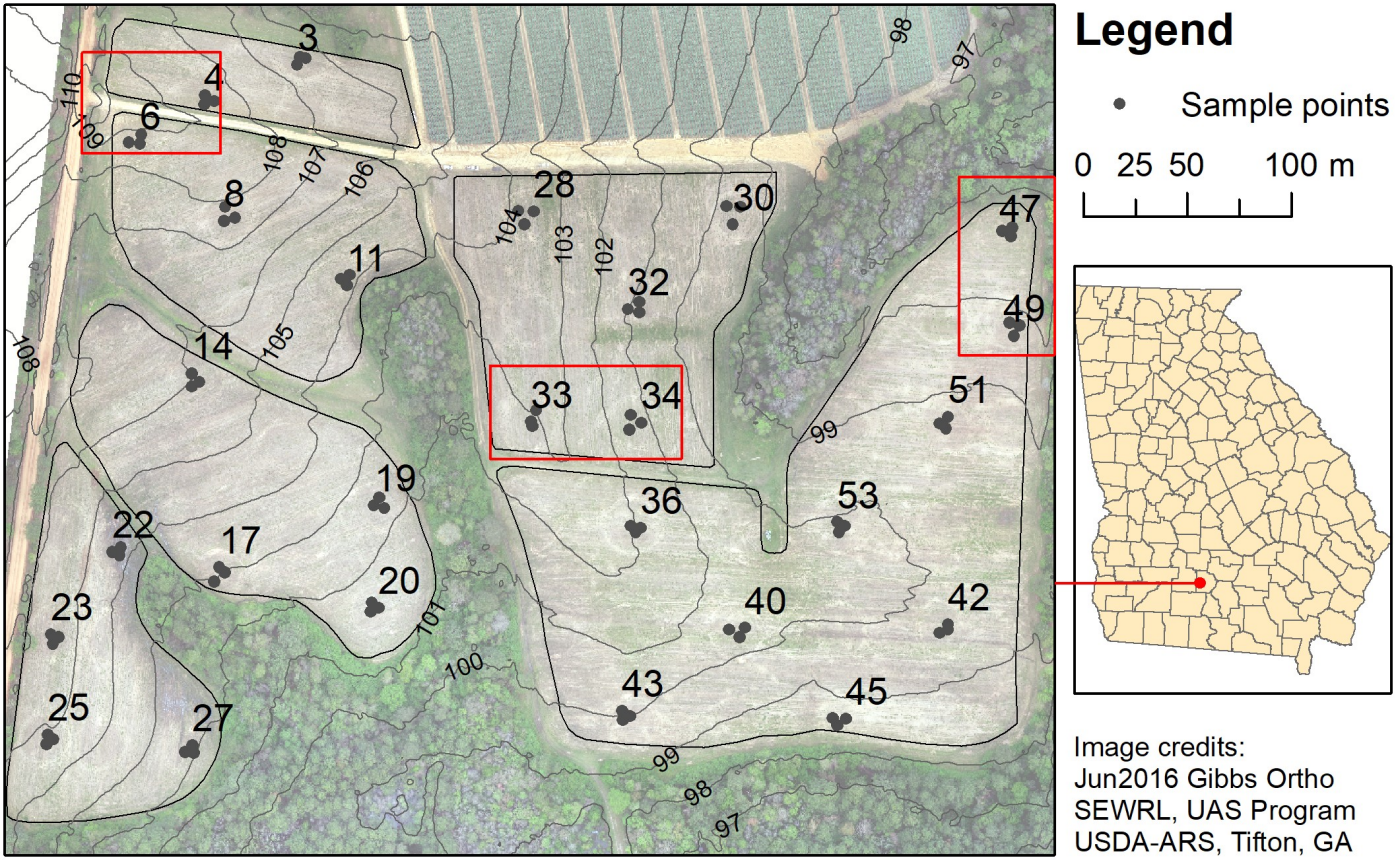
Map showing location and configuration of study area, and sample points. Main points labeled by number. Red boxes indicate locations of main points with detailed data visualized in S7 Fig.

The study was conducted in 2015 and 2016 in the Southeastern Plains ecoregion of Georgia, USA [43] located near TyTy, GA (31.44° N, 83.59° W) within a 13.84 ha area planted in 2013 to a bioenergy feedstock grass, *Miscanthus x giganteus* (*Miscanthus×giganteus* Greef and Deuter ex Hodkinson and Renvoize), referred to here as *M*. *giganteus*, in 2013 (Fig 1). Lime was applied to the field at 1121 kg/ha (24% Ca and 8% Mg) on 4 March 2015 and at 2245 kg/ha on 24 June 2016. Fertilizer was applied (35:04:09 NPK pretreated with Agrotain^®^) on 23 March 2015 (238 kg/ha) and 22 June 2015 (238 kg/ha), 22 March 2016 (224 kg ha^-1^), 8 April 2016 (705 kg ha^-1^) and 22 June 2016 (793 kg ha^-1^). A grid of points (n = 73, approx. 40 m apart) was previously established to collect above ground biomass and soil cores [32]. Within this grid, 27 points were randomly selected (referred to here as “main” points) to measure specific environmental parameters and insect and spider abundance, and 3 sample locations were established, clustered at each main point, for a total of 81 sample locations. Arthropods and NDVI were sampled 3 times each year: on 6 July, 3 August, and 31 August in 2015; and 6 July, 3 August, and 7 September in 2016. Hereafter the collection dates are referred to as “early”, “mid” and “late” seasons, the first, second and third collections respectively, each year.

### Canopy dwelling insect sampling

**Fig 2:**
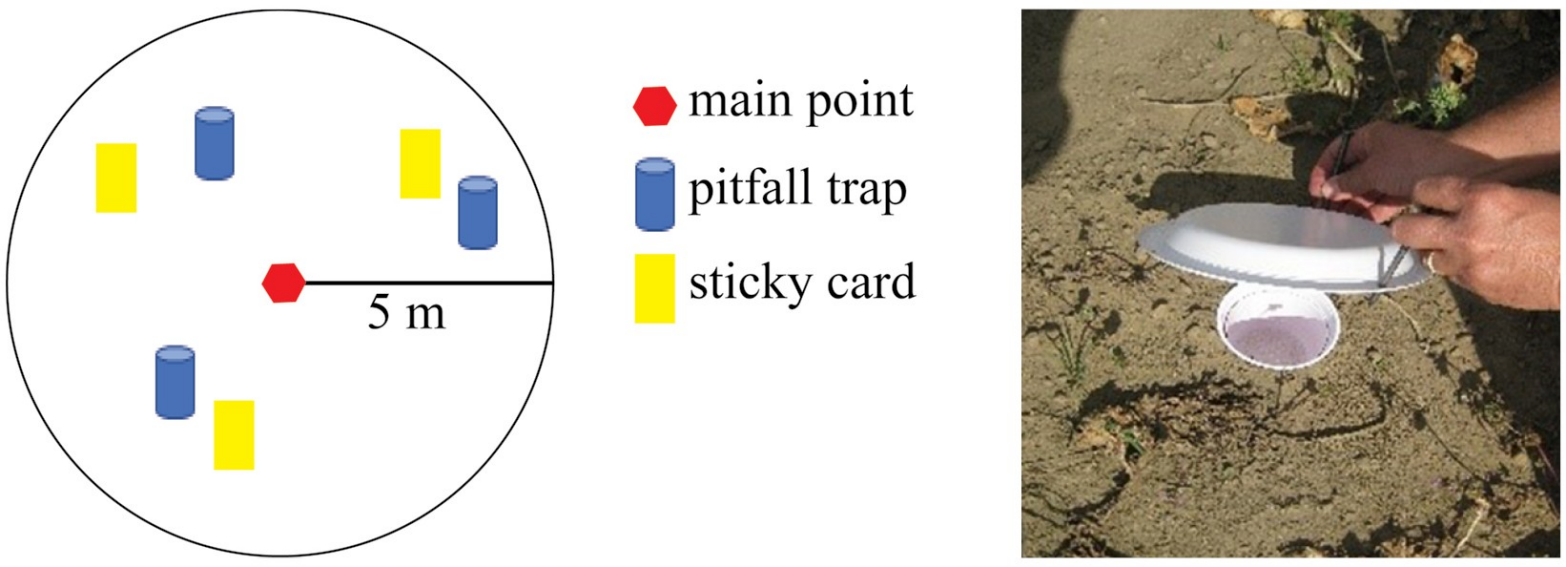
A. Diagram of sampling strategy. Main point indicates location within field (see Fig 1). B. Photo of pitfall trap set up.

At each main point, 3 insect yellow sticky card traps were located within a 5 m radius sampling area by dividing the circle into thirds and randomly placing a trap within that area (Fig 2A). The traps consisted of 7 × 12 cm sticky cards (Olson Products, Medina, Ohio USA) that were placed so that the top of the card was level with the top of the crop canopy and this position was maintained throughout the season in both years. Once placed, sticky card trap locations were recorded using a Trimble Geo7X GPS instrument with a ± 50 cm error. Sticky traps were placed in the field for a one-week period and collected at each sampling date. Insect predators and parasitoids were pooled as insect natural enemies, and aphids, whiteflies and thrips were pooled as phloem-feeding species for the analyses. Thrips, aphid, and whitefly densities on sticky traps were estimated by counting all adults and larvae within two 2.0 by 2.0 cm randomly selected areas on each side of the card (16 cm^2^ per card). All other insects were estimated by counting all individuals on both sides of the card (168 cm^2^ per card). In 2016, year two of the study, a GPS unit was used to replace traps to within 1 m of the 2015 location.

### Ground dwelling spider sampling

To estimate ground dwelling spider populations, pitfall traps were collected from the same locations as the sticky cards. Pitfall traps consisted of two 16 oz plastic cups (Solo Cup Company, Lake Forest, Illinois, USA) with the outer cup tightly installed in a hole in the ground after removing the bottom of the cup. The upper edge of the inner cup was removed; a 1% Tween^®^ solution with salt was added, and the cup was installed within the outer cup in the ground. To reduce plant and other materials falling into the cup, a cover was installed and secured with nails at three points to allow an approximately 3 cm opening above the ground (Fig 2B). Traps remained in the field for a one-week period and were collected on the same dates as the sticky cards. Collected spiders were counted, recorded, and transferred to plastic containers containing 80% ethanol. The spider families identified included Gnaphosidae, Linyphiidae, Phrurolithidae, Lycosidae, Oxyopidae, Salticidae, Thomisidae, Araneidae, Tetragnathidae, Agelenidae, and Corrinidae.

### Environmental variables

Multiple environmental explanatory variables were estimated at each sample point over the course of the season (Fig 1; Table 1). Environmental factors included: elevation (ELEV), percent slope (SLOPE), aspect, normalized difference of vegetation index (NDVI), distance to the nearest woodland edge (DISFOR), several weather values, and soil components (i.e. percent sand, clay and silt at 0–15 cm, with and without gravel component). Elevation (ELEV) and SLOPE at each sample point were measured from gridded raster datasets derived from the 2016 digital elevation model for Tift County, GA [44], which provides elevation in meters above sea level. The original data were reprojected from geographic coordinates to the Universal Transverse Mercator projection (Zone 17 North) converting horizontal measurements to meters and resampled to 1-meter pixel resolution. The data, originally consisting of four tiles were mosaicked to produce a seamless dataset, then a mask was applied to extract the values within the watershed surrounding the study area. The elevation data were then used to derive a slope map, where slope is the first derivative of the elevation map, calculated as the rate of change from one pixel to the next, expressed as degrees (0 to 360). Aspect was likewise calculated from the elevation map, and a traditional planar method was used to calculate the compass direction (0 to 360 degrees) of the downhill slope [45]. To use aspect in the models, degrees were converted to radians and transformed into axial components with values ranging from -1 to 1 along each cardinal axis. Thus, southward facing aspect, or southward index (SI), was computed as cos(aspect), where values of -1 to 1 indicate southward to northward facing slopes. Likewise, westward index (WI) was computed as sin(aspect), where -1 to 1 indicate westward to eastward facing. To test for importance of landscape position, Euclidian distance to the nearest forest edge (DISTFOR) was calculated.

**Table 1:** Description of predictor variables used in initial regression analyses.

Regression Variable	Description
ELEV	Elevation (meters above mean sea level)
SLOPE	Percent slope
SI^a^	Southward facing aspect, southward index
WI^b^	Westward facing aspect, westward index
DISTFOR	Distance to forest edge (meters)
CLAYNR	Percent clay (gravel removed)
SILTNR	Percent silt (gravel removed)
SANDNR	Percent sand (gravel removed)
GRAVEL	Percent gravel
CLAY	Percent clay
SILT	Percent silt
SAND	Percent sand
CUMGDD	Cumulative growing degree days (starting DOY 84; base temp = 6 deg. C)
WKAVEWINDSP	Average wind speed (miles/hour) for 7 days prior to including sample date
WKAVETEMP	Average air temperature (degrees C.) for 7 days prior to and including sample date
WKAVEMINRELHUM	Average relative humidity (percent) for 7 days prior to and including sample date
WKAVEPRECIP	Average precipitation (mm/day) for 7 days prior to and including sample date
WKAVEWINDDIR_SI^c^	Average wind direction, southward index
WKAVEWINDDIR_WI^d^	Average wind direction, westward index
NDVI	Normalized difference of vegetation index [–1, 1]
INS_NET_1	Total count of natural enemies with gap filling on missing values
SPI_TOT_1	Total count of spiders with gap filling on missing values
INS_PHLOEM_1^e^	Total count of phloem feeders with gap filling on missing values

^a^ SI = cos(aspect), [–1, 1]; southward to northward facing.

^b^ WI = sin(aspect); [–1, 1]; westward to eastward facing.

^c^ WKAVEWINDDIR_SI = cos(wind direction, radians), [–1, 1]; southward to northward blowing.

^d^ WKAVEWINDDIR_WI = sin(wind direction, radians), [–1, 1]; westward to eastward blowing.

^e^ Response variable.

Seven-day averages were calculated for weather measurements (temperature, wind speed, wind direction, relative humidity and precipitation) obtained from a Southeast Watershed Research Lab weather station located on the adjacent University of Georgia experimental farm [46]. Like the aspect variable, wind direction was decomposed into two variables to provide a continuous range of values of -1 to 1. Seven-day averages were also calculated for cumulative growing degree days (CUMGDD) for each of the six sampling dates starting on Day of Year (DOY) 84 (March 25/24, 2015/16), using a base temperature of 6°C [47].

The Normalized Difference of Vegetation Index (NDVI), a spectral vegetation index that detects the greenness of vegetation, was measured at each sample point. The index is calculated as the difference of reflected near infrared (NIR; 0.75–1.4 μm) and red light (0.63–0.74 μm), normalized by their sum [NDVI = (NIR-RED) / (NIR+RED)] [48], and is related to photosynthetic activity in plants and crop yield [49]. In agriculture, NDVI is routinely used in soil nutrient monitoring, for crop fertilization and irrigation recommendations, and for monitoring plant stress, which can be related to insect damage, drought, or nitrogen deficiencies, and is indicated in NDVI anomalies. For this study, NDVI was measured at each field location using a handheld Trimble Greenseeker^®^, a trigger-activated sensor that illuminates the crop canopy with red and NIR light and provides instantaneous values of NDVI calculated from the measured backscatter. To record average values, operators must depress the trigger for the entire period of measurement. For this study, field technicians suspended the instrument ~50 cm over the crop canopy at arm’s length and rotated in a circle with the sensor activated for ~10-second intervals. The area recorded by the sensor therefore constituted an average of crop canopy values for a circle ~2.5 m in diameter. Readouts from the instrument were manually recorded on the same dates when sticky cards were collected.

Soil at the research site is predominantly classified as a Tifton loamy sand with smaller areas comprised of a Carnegie sandy loam [50, 51]. Soils in this region commonly contain plinthite layers formed via repeated redox cycles resulting in Fe concentration and formation of partially cemented subsoil layers that lead to perched water tables and lateral subsurface flow [52–54]. Soils were sampled in 2017, across a grid of points, including the points used in this study (methods described in S6 Fig), and the percent of soil sand, silt and clay at a depth of 0–15 cm was determined [55] both with and without the gravel fraction.

### Statistical analysis

#### Arthropod field data

ANOVA was used to evaluate the overall effect of year, season (early, mid-, and late) and their interactions on the density of phloem feeders, insect natural enemies and spiders with Tukey’s HSD used to separate the means [56].

#### Data interpolation and harmonization

Because this study involved the use of multiple data types collected by several investigators, the analysis required data harmonization involving their transformation from disparate data sets into one cohesive data set. Transformations included interpolation of values and offsetting locations for analysis.

Insect and spider sample values in both sticky card and pitfall traps included missing values due to missing traps and transport loss that were recorded as null (INS_PHLOEMT and INS_NET, Null = 16; SPI_TOT, Null = 25). However, a null data value at those locations would eliminate the use of all data at these points in the GWR. To maximize the data use and accomplish the GWR, gaps in the data were filled by averaging values of nearest neighbors within 100 m (number of neighbors provided in results data table), which was over twice the average distance between main points in the field. Subsequently, the gap-filled data were used for all regression analyses. The dataset included two years of samples collected at the same locations.

As noted, the arthropod sampling grid was a subset of a larger grid from which soils were sampled and measured. However, while the soil cores were collected at the main points, arthropod sample points were within a 5 m distance of those locations. To harmonize the data, a kriging function was used to interpolate soil values, producing a raster data layer (2 m pixel resolution), and values were extracted from the raster at each of the sample points. This produced a set of seven soil maps, one for each of the soil fractions, both with and without gravel content (S6 Fig).

#### Regression analysis

To analyze and understand the relationships between herbivorous insect pests, environmental variables and their insect and spider predators, numerous multiple regression models were used. Data exploration began with generalized linear models of the entire dataset, and then proceeded into three partitioned groups (early, mid, and late season). Local effects and how relationships changed in space and over the course of the growing season were examined in more detail using GWR. Esri ArcGIS^®^ desktop software was used for all data processing and spatial statistical analysis, using scripts available in the ArcGIS Pro Spatial Statistics toolbox, and the Spatial and Geostatistical Analyst extension modules.

Exploratory regression of environmental variables and phloem feeder trap counts suggested a subset of variables that were effective predictors of their counts, which were incorporated into an Ordinary Least Squares (OLS) regression model. Various diagnostics were used to evaluate and select best fitting models. Adjusted R-Squared (AdjR2) and corrected Akaike Information Criterion (AICc) measured overall global fit [57], Global Moran’s I p-value (SA) indicated spatial autocorrelation in the residuals of the regression models, while the variance inflation factor (VIF) was used to diagnose conditions of multicollinearity among variables in the models [58]. An approach to model development was used that maximized the search potential of the exploratory regression tools by examining a maximum of 15 out of 19 potential explanatory variables. Model cutoff limits were set to limit the number of passing models using criteria set for diagnostics of min AdjR2 >0.10, max coefficient p-value < 0.20, max VIF <7.50, and min SA >0.05. Initially AdjR2 and AICc were used to evaluate candidate models. To further define a working OLS model, regressions were carried out and model coefficients, magnitudes, and direction of effects on the response variables were examined. Through this process optimal models were identified for the global dataset and for seasonally grouped datasets, minimizing AICc, spatial autocorrelation and multicollinearity, and identifying a subset of explanatory variables [58].

To explore the locally varying influence of terrain, soils, vegetation, and insect and spider predators on phloem feeders, an iterative approach was used to identify meaningful GWR models. GWR builds location specific regression models and the actual locations of the field samples were assumed to be within one meter of each other from one year to the next. However, because all six observations (3 season x 2 years) were mapped to the same coordinates, producing “multipoint” data the software would truncate all year two observations when data were partitioned by season. To avoid this problem, the coordinates of the six coincident points were offset by 0.5 m in cardinal directions—well within the measurement error of the field observations, effectively “exploding” the multipoint feature, and thus allowing the use of all data in the GWR.

Beginning with the best fitting model (i.e. lowest AICc) developed for each seasonal group, GWR was used to further explore and refine the regression models. GWR draws from neighboring values to build a local regression model, so the neighborhood search and weighting scheme parameters used are important factors in determining the model results. For these analyses, model parameters included accounting for the data type (count) with a Poisson distribution, and a neighborhood selection function that iteratively includes increasingly distant neighbors until the AIC value for the model is optimized. A “Bisquare” local weighting scheme for the GWR kernel density decay function was used, so that remaining features had no influence on the local regression function beyond the neighborhood distance value of the optimized model. The Bisquare scheme was selected over a Gaussian weighting scheme as more appropriate to identifying features impacting the distribution of phloem feeders within a field. In addition to using AICc to evaluate and compare models, GWR model diagnostics that measure goodness of fit were used to assess how well the local models performed compared with a global “non-spatial” model. Specifically, these included the percent of deviance explained by the global model, the local model, and the local vs. global model. Through this process GWR models for each season were identified with optimal AICc values, each of which were an improvement over global, non-spatial regression models. These optimal models resolved to a common set of variables. However, because one terrain-related variable alternated between ELEV and SLOPE across the seasons, both variables were included in the final models with slight improvements to goodness-of-fit measures. Evaluation of coefficients in GWR is currently an area of active research with no clear consensus on best methods. Consequently, the ratio of the coefficient to the standard error (C:SE) was used to evaluate the magnitude of coefficients relative to their variability, and to map the relative stability of coefficient values, termed “credence” for this analysis.

#### Data visualization

Visualization of the data allowed for the comparison of results using mapping displays. Soils, terrain, and the coefficients from GWR models were interpolated and displayed. Mapped surfaces of coefficient values were overlaid with values of estimated credence. To properly display the surfaces and values together, a geometric classification scheme was used to symbolize coefficient raster surfaces, while estimations of credence (i.e. C:SE) were symbolized as points, using a manual classification, relying on Chebyshev’s theorem [59] to indicate values of: high levels of credence (<-3; >3), some credence (-3 to -2; 2 to 3) and little credence (>-2 to <2). Color-coded graphics were used to compare changes over time in both the study area, broadly, and in the values of specific points. The visual inspection of maps and charted data provided interpretable figures from which conclusions could be drawn.

## Results

### Arthropod field data

Arthropod samples were counted, summarized by family, and grouped by functional guild. Spider diversity information was also summarized using the Shannon diversity index. Phloem feeders observed included thrips spp. (Thysanoptera), aphid, and whitefly spp. (Hemiptera). Generalist predator species included *Orius* spp. (Hemiptera: Anthocoridae), *Geocoris* spp. (Hemiptera: Lygaeidae) and *Coleomegilla maculata* De Geer, *Harmonia axyridis* Pallas, *Hippodamia convergens* Guérin-Méneville, *Coccinella septempunctata* Linnaeus and *Scymnus* spp. (Coleoptera: Coccinellidae). Parasitoids included the aphid parasitoid, *Lysiphlebus testaceipes* Cresson (Hymenoptera: Braconidae). Detailed arthropod data are provided in the associated online data table [60].

ANOVA results showed that there was no interaction between year (2015, 2016) and season (early, mid, and late) on the density of phloem feeders (F_2/463_ = 2.12, P = 0.121). There was a significant effect of year (F_2/463_ = 5.75, P = 0.017) and season (F_2/463_ = 99.90, P < 0.001) on the density of phloem feeders. Phloem feeder density was higher in 2016 than in 2015 and higher early season than mid-and late season both years (Table 2). There was an interaction between year and season on the density of insect natural enemies (F_2/462_ = 78.79, P < 0.001) and spiders (F_2/455_ = 16.79, P < 0.001). In 2015, the density of insect natural enemies was highest mid-season and lowest early season, whereas in 2016 their density was highest early season and lowest late season (Table 3). In 2015, the density of spiders increased over the season, whereas in 2016 their density was highest mid-season and lowest early season (Table 4).

**Table 2:** Density of herbivorous insects per cm^2^ area of sticky card early, mid, and late season in 2015 and 2016.

Year	Season	Mean ± SE (n)
2015	Early	30.68 ± 2.80 (73) a
	Mid	11.47 ± 1.05 (78) b
	Late	4.87 ± 036 (77) c
2016	Early	35.67 ± 2.35 (81) a
	Mid	10.85 ± 2.54 (81) b
	Late	11.85 ± 1.11 (81) b

Different letters within the column are significantly different a P < 0.05.

**Table 3:** Density of insect natural enemies per cm^2^ area of sticky card early, mid, and late season in 2015 and 2016.

Year	Season	Mean ± SE (n)
2015	Early	0.67 ± 0.11 (81) d
	Mid	5.82 ± 0.59 (81) a
	Late	3.34 ± 0.39 (77) b
2016	Early	4.59 ± 0.37 (81) a
	Mid	1.27 ± 0.17 (81) c
	Late	1.16 ± 0.17 (80) c

Different letters within the column are significantly different a P < 0.05.

**Table 4:** Density of spiders per pitfall trap early, mid, and late season in 2015 and 2016.

Year	Season	Mean ± SE (n)
2015	Early	4.90 ± 0.78 (72) c
	Mid	7.69 ± 0.49 (78) b
	Late	8.85 ± 0.58 (75) a
2016	Early	1.57 ± 0.18 (81) d
	Mid	9.39 ± 0.62 (74) a
	Late	4.71 ± 0.49 (81) c

Different letters within the column are significantly different a P < 0.05.

### Environmental variable data

Terrain and soils data were interpolated from a digital elevation model, and soil core results (S6 Fig), respectively, and are summarized in Table 5 (maps for topography, slope, aspect and soils, are provided in S1–S3 Figs, and S6A–S6G Fig). Study area elevations ranged from 97 meters above mean sea level (mamsl) to 109 mamsl with the average elevation at 102.54 mamsl. The terrain was typical of the region, with slopes ranging from less than one percent to almost seven percent, averaging about 3.5 percent, and predominantly southeastward facing with the average SI of -0.23, and WI of 0.53. The field shapes were irregular (as seen in Fig 1) and surrounded on most sides by forest. Distance from sample points to the forest edge was about 35 m but was highly variable and ranged from 6 to 81 m. Soils in the field had widely varying fractions of gravel, but at most locations soils were predominantly sand with smaller fractions of silt and clay.

**Table 5:** Summary of terrain and soil variables, n = 81.

Variable name	Variable description	Min	Max	Mean ± SE
ELEV	Elevation (meters above mean sea level)	97.23	108.88	102.54 ± 3.10
SLOPE	Percent slope	0.60	6.87	3.46 ± 1.35
SI^a^	Southward index [–1, 1]	-1	1	-0.23 ± 0.648
WI^b^	Westward index [–1, 1]	-0.89	1	0.53 ± 0.487
DISTFOR	Distance to forest, m	5.84	80.53	34.91 ± 19.94
CLAYNR	Percent clay	1.82	13.29	4.89 ± 2.39
SILTNR	Percent silt	22.44	36.43	31.29 ± 2.70
SANDNR	Percent sand	50.22	77.23	63.67 ± 4.61
GRAVEL	Percent gravel	4.98	82.06	18.74 ± 15.35
CLAY	Percent clay (incl. gravel)	<0.01	7.70	3.77 ± 1.46
SILT	Percent silt (incl. gravel)	7.24	34.41	24.88 ± 5.22
SAND	Percent sand (incl. gravel)	11.14	74.13	52.62 ± 11.52

^a^ SI = cos(wind direction, radians), [–1, 1]; southward to northward facing.

^b^ WI = sin(wind direction, radians), [–1, 1]; westward to eastward facing.

In general, warm, calm, and humid weather conditions, typical of this region [61], occurred at the site leading up to and during sample collection periods (Table 6). During these dates, temperatures averaged 25 to 27 degrees C, winds averaged 1.6 to 4.1 miles per hour (mph), and relative humidity 44 to 54 percent. Winds were predominantly blowing southward but shifted between slightly westward to slightly eastward. Based on the cumulative growing degree day value (CUMGDD) *M*. *giganteus* crop development was further advanced in 2015 during early, mid, and late season samples, with CUMGDD ranging from 1874 to 3122 in 2015, compared with 1812 to 3191 in 2016. Values for NDVI (Table 7) were higher at the early season sample date in 2015 than 2016, averaging 0.65 versus 0.40, respectively. These characteristics were reversed for late season values, when NDVI averaged 0.44 and 0.66 for 2015 and 2016, respectively.

**Table 6:** Summary of weather variables, early, mid, and late season in 2015 and 2016.

Year	Season	CUMGDD	Ave Wind Speed (mph)	Ave Temp (deg C)	Ave Rel Hum (percent)	Ave Precip (mm/day)	Ave Wind Dir SI^a^	Ave Wind Dir WI^b^
2015	Early	1874	2.89	25.1	54.0	7.1	-0.76	-0.62
	Mid	2514	1.60	27.5	48.2	7.4	-0.75	0.33
	Late	3122	1.73	25.2	48.8	1.5	-0.65	0.35
2016	Early	1812	3.74	27.3	48.6	5.2	-0.75	-0.57
	Mid	2439	3.09	27.6	43.5	12.5	-0.76	-0.59
	Late	3191	4.13	24.9	46.7	14.9	-0.53	0.42

Average values are based on the average daily values for 7 days prior to and including sample date. Wind speed was measured in miles per hour (mph). CUMGDD is cumulative growing degree days (starting on DOY84, base temp 6°C).

^a^ Wind Dir SI = cos(wind direction, radians), [–1, 1]; southward to northward blowing.

^b^ Wind Dir WI = sin(wind direction, radians), [–1, 1]; westward to eastward blowing.

**Table 7:** Summary of normalized difference of vegetation index (NDVI), n = 81, early, mid, and late season in 2015 and 2016.

Year	Season	Min	Max	Mean ± SE
2015	Early	0.38	0.83	0.65 ± 0.09
	Mid	0.14	0.76	0.57 ± 0.07
	Late	0.25	0.73	0.44 ± 0.11
2016	Early	0.31	0.54	0.40 ± 0.05
	Mid	0.37	0.76	0.64 ± 0.10
	Late	0.35	0.80	0.66 ± 0.10

### Exploratory regression of global models and Ordinary Least Squares (OLS) results

Analysis began with an exploration of non-spatial, global models of responses of phloem feeders to both environmental variables and predators (n = 486). Only a subset of the 490,935 regression trials passed cutoffs of model diagnostics. Lowest AICc values were found for a model with 10 of 19 possible variables at 4039.84 (Model 1, Table 8). More parsimonious models were also found to be within 3 AICc points, considered to be comparable, so models up to 4042.84 were also reviewed. Several models with 9 or 8 explanatory variables came into this category. However, in all cases VIF values were >7.5, indicating multi-collinearity existed in these models, so those with the lowest values were selected to explore further. All models also had high SA values, indicating spatial autocorrelation was present. Looking toward a more parsimonious approach, regression models with fewer variables (Models 2 and 3, Table 2) were further explored.

**Table 8:** Regression model diagnostic values for selected models of phloem feeder abundance.

Model number	No. variables	AdjR2	AICc	VIF	SA	Model
1	10	0.42	4039.84	26.68	0.38	-ELEV***, -SILTNR***, -SANDNR***, -GRAVEL**, -SILT**, -NDVI***, -WKAVEWINDDIR_SI, +WKAVEWINDSP***, +WKAVETEMP**, +WKAVEMINRELHUM***
2	9	0.42	4041.25	8.86	0.76	-ELEV***, -SILTNR***, -SANDNR***, -NDVI***, -WKAVEWINDDIR_SI*, +WKAVEWINDSP***, +WKAVETEMP**, +WKAVEMINRELHUM***, -SPI_TOT_1
3	8	0.42	4041.39	8.84	0.60	-ELEV***, -SILTNR***, -SANDNR***, -NDVI***, +WKAVEWINDSP***, +WKAVETEMP***, +WKAVEMINRELHUM***, -INS_NET_1

Model 1 provided best fit according to model diagnostics, but models 2 and 3 were comparable and more parsimonious. AdjR2 is adjusted R-Squared; AICc is corrected Akaike’s Information Criterion; VIF is variance inflation factor; SA is Global Moran’s I p-value; model variable sign is plus or minus (+/-); model variable significance is indicted by asterisks (* = 0.10; ** = 0.05; *** = 0.01).

In models 2 and 3, predator (SPITOT_1 and INS_NET_1) covariates were not significant, and the magnitudes and directions of effects of explanatory variables in both models were very similar. In both models, SAND_NR had high VIF values (~8.8) as did WKAVETEMP (7.3 and 7.9). Furthermore, all soil and weather variables showed high levels of multicollinearity.

To reduce multicollinearity in OLS models, variables were removed one by one. Model diagnostics of AdjR2, AICc, Koenker statistic and the Joint Wald statistic (JWald), and variable diagnostics of p<0.01, and VIF<7.5, were used to compare results and identify best fitting OLS models. By this iterative process, a pair of models were selected, one with six variables, and one with four (Models 4 and 5, respectively, Table 9). While model 4 provided a better diagnostic fit according to AdjR2 and AICc, model 5 better describes the hypothesized drivers that determine the abundance and distribution of phloem feeders.

**Table 9:** Global Ordinary Least Squares (OLS) models optimized (model 4) and hypothesized (model 5).

Model number	Parameters	Coeff (Robust_SE)	Model Diagnostics
4	Intercept	-319.36 (56.74)*	AdjR2 = 0.38
	-ELEV	-125.29 (137.52)*	
	-SILTNR	-480.99 (46.45)*	
	-NDVI	-26.58 (5.78)*	AICc = 4079.22
	+WKAVEWINDSP	9.55 (0.70)*	JWald = 276.93* (df6)
	+WKAVETEM	6.10 (.052)*	Koenker = 22.3* (df6)
	+WKAVEMINRELHUM	3.59 (0.34)*	
5	Intercept	59.61 (15.68)*	AdjR2 = 0.20
	-SILTNR	-102.89 (48.36)*	
	-NDVI	-32.44 (6.46)*	AICc = 4196.76
	+WKAVEWINDSP	4.75 (0.75)*	JWald = 131.4* (df4)
	-SPI_TOT_1	-0.89 (0.15)*	Koenker = 11 (df4)

Model 4 provides the optimized model for phloem feeder abundance based on diagnostics. Model 5 provides the best fitting model adjusted to include hypothesized drivers of phloem feeder abundance and distribution. AdjR2 is adjusted R-Squared; AICc is Akaike’s Information Criterion; JWald is Joint Wald Statistic; model variable sign is plus or minus (+/-); coefficient and Statistic significance are indicted by asterisks (p<0.01); df indicates degrees of freedom. Response variable was INS_PHLOEMT_1 (n = 486) for all models.

#### Weather variable results

**Fig 3:**
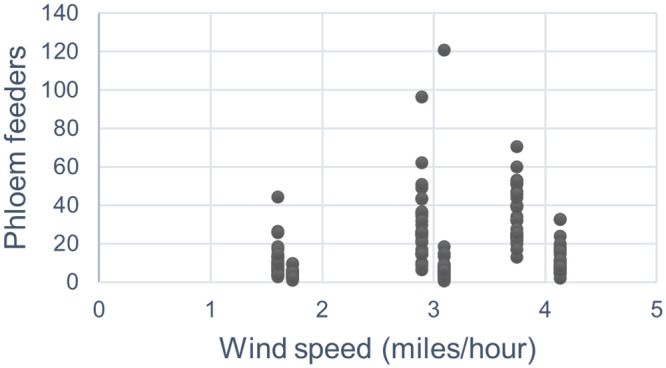
Scatterplot of number of phloem feeder and average 7-day windspeed for six dates in 2015 and 2016.

Weather variables of seven-day average wind speed, relative humidity and average temperature all showed positive correlations with phloem feeder abundance in global models. However, VIF values for these variables in Model 2 were 1.48 to 7.88, indicating multicollinearity, so all but wind speed with lowest VIF, were dropped in the best-fitting models (i.e. Models 4 and 5). In subsequent models, broken out by season, weather factors were not included because there was only one value per sample date, or 2 per season, and the values measured were constant for all locations. Nevertheless, in global models, the relationship between phloem feeders and wind speed was positive. Phloem feeders were most abundant at wind speeds of 2.89 mph and 3.74 mph (Fig 3), which appear related to early season values, but without more time-series data, seasonal determinations of weather effects are inconclusive.

#### Model selection using seasonal data

Although the data did not lend themselves to a full time series analysis, we investigated the effects of seasonality in the early, mid, and late season groups, corresponding with the first, second and third collections each year (n = 162 for each group). Exploratory regression indicated that seasonal models were better fitting than the global OLS models (Table 10) where, most notably AICc values were much smaller. However, measures of multicollinearity (VIF values) remained high, especially for the mid-season model, likely due to the interaction between soil texture and water availability in the context of two very different precipitation regimes captured during the two-year study. The Joint Wald (JWald) statistic indicated seasonal models were, overall, significant. In addition, the Koenker test statistic for mid- and late season models was statistically significant (p<0.01), potentially indicating non-stationarity or heteroskedasticity, and suggesting that a spatial model could be an improvement.

**Table 10:** Best fitting regression models for phloem feeder abundance early, mid, and late season, model parameters and diagnostics.

Season	Parameters	Coeff (Robust_SE)	Model Diagnostics
Early	Intercept	568.53 (105.33)*	AdjR2 = 0.26
	-SILTNR	-696.28 (137.52)*	AICc = 1417.95
	-SANDNR	-480.99 (99.88)*	VIF = 4.37
	-NDVI	-16.71 (10.58)	JWald = 34.4* (df4)
	-SPI_TOT_1	-0.87 (0.51)	Koenker = 8.66 (df4)
Mid	Intercept	1137.84 (233.64)*	AdjR2 = 0.59
	-ELEV	-3.61 (0.66)*	
	-WI	-4.49 (1.57)*	
	-SILTNR	-788.16 (200.33)*	AICc = 1250.37
	-SANDNR	-598.66 (138.85)*	VIF = 31.51
	-GRAVEL	-96.99 (33.57)*	JWald = 40.2* (df8)
	-CLAY	-481.61 (151.91)*	
	-SILT	-248.28 (90.26)*	
	-NDVI	-47.07 (13.11)*	Koenker = 44.9* (df8)
Late	Intercept	35.34 (7.40)*	AdjR2 = 0.26
	+SLOPE	0.82 (0.38)*	
	+CLAYNR	105.53 (45.09)*	AICc = 1097.13
	-GRAVEL	-30.53 (9.05)*	VIF = 3.55
	-SILT	-102.63 (24.62)*	JWald = 38.26* (df6)
	-INS_NET_1	-0.65 (0.23)*	Koenker = 15.7* (df6)
	-SPI_TOT_1	-0.34 (0.09)*	

For seasonal regression models, weather variables were excluded, and soils variables were significant in all models. The mid-season model reported here were best fitting by model diagnostics, however, high VIF values indicate high levels of multicollinearity among soil variables. AdjR2 is adjusted R-Squared; AICc is corrected Akaike’s Information Criterion; JWald is Joint Wald Statistic; model variable sign is plus or minus (+/-); coefficient and Statistic significance are indicted by asterisks (p<0.01); df indicates degrees of freedom. Response variable was INS_PHLOEMT_1 (n = 162) for all models.

### Geographically weighted regression results

**Fig 4:**
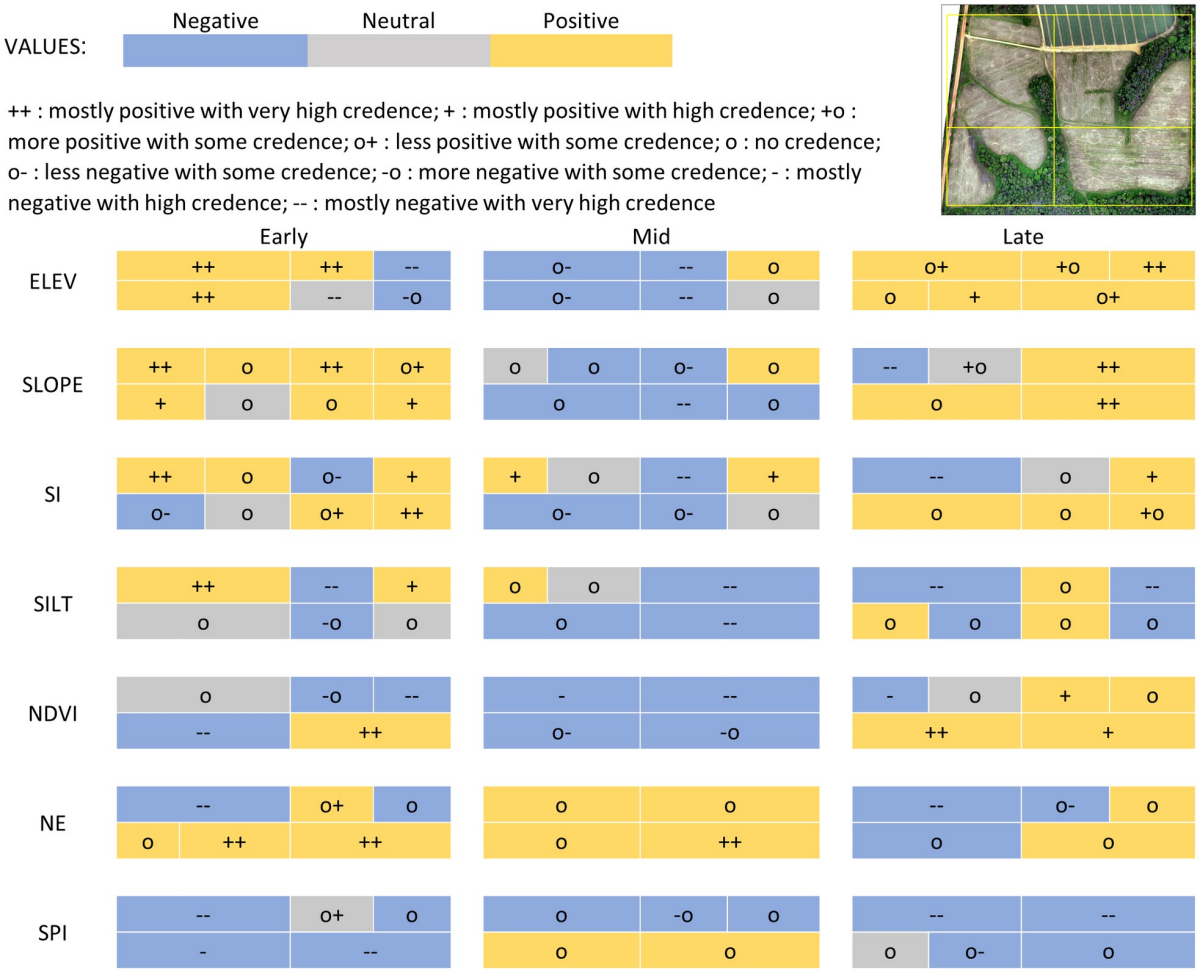
Heat map of geographically weighted regression (GWR) coefficient signs as related to map surfaces across fields. Early, Mid and Late columns refer to sample dates. Rows refer to explanatory variables. Columns are divided into four quadrants corresponding roughly to four regions of the fields as shown in the inset map. In some cases, a quadrant is further subdivided, but this is only to indicate that there was no single positive trend across that quadrant.

To explore the locally varying influence of terrain, soils, vegetation, and predators on phloem feeders, geographically weighted regression (GWR) was used to identify regression models at a local level. Elements of multicollinearity were removed by testing the influence of soil variables in isolation, and iteratively testing each group for each date. Through this process GWR models for each season were identified with optimal AICc values, where the optimizing neighborhood distance routine resolved on a distance band of ~169 m, indicating the search distance where the GWR model was optimized. Finally, a common model was identified, which allowed for the comparison of coefficient values and trends across all three seasons. The seven explanatory variables that influenced phloem feeder spatial distribution included: ELEV, SLOPE, SI, NDVI, SILTNR, INS_NET_1, and SPI_TOT_1. All GWR models thus specified were an improvement over global, non-spatial regression models; diagnostics of the percent deviance explained (i.e. the proportion of explanatory variable variance accounted for by the model) indicated that local models were better fitting than global models (Table 11). GWR results, summarized by field region (Fig 4), showed that during the growing season, the relationship between these environmental variables and phloem feeders changed over space and time, but credence in coefficient values was often high, whether the coefficient was positive or negative. A closer look at detailed GWR results suggested that responses to variables may be clustered in regions of the field, indicating a potential for future analysis identifying zones of similarity in environmental and predator dynamics and phloem feeder responses (S7 Fig). One important caveat is that the GWR algorithms are implemented in several software packages (e.g., R), but for this analysis ArcGIS Pro (v 2.5) Spatial Statistics toolbox was used, which calculates parameter estimates using a spatial weighting function that may differ from other packages (https://pro.arcgis.com/en/pro-app/tool-reference/spatial-statistics/how-geographicallyweightedregression-works). As a result, parameter estimates provided in our analyses may not match those from other GWR implementations.

**Table 11:** Model diagnostics of geographically weighted regression (GWR) models for early, mid-, and late season (n = 162).

Season	Distance Band (m)	Global dev	Local dev	Loc v Glob	AICc
**Early**	168.7	0.2341	0.5294	0.3856	1063.04
**Mid**	169.6	0.6781	0.7903	0.3486	552.10
**Late**	168.9	0.3200	0.6076	0.4230	476.63

All models included ELEV, SLOPE, SI, SILTNR, NDVI, INS_NET_1, and SPI_TOT_1 as explanatory variables and response variable was INS_PHLOEMT_1. Global dev is percent global deviance explained by the non-spatial global model (0.0 to 1.0); Local dev is the percent deviance explained by the local (GWR) model (0.0 to 1.0); Loc v Glob compares the residual sum of squares of the local to that of the global model (0.0 to 1.0), with higher values indicating the GWR model was better fitting. AICc is the corrected Akaike Information Criterion.

#### Elevation (ELEV)

**Fig 5:**
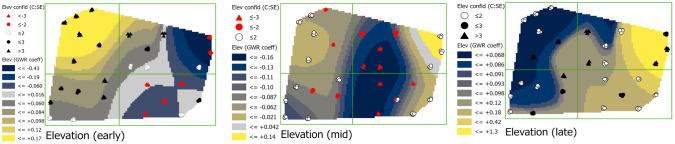
Maps showing early, mid-, and late season elevation (ELEV) coefficients with estimations of credence in coefficient values. Coefficient surface values were symbolized using a geometric interval classification scheme from blue to gold, with grey indicating the interval including zero (if present).

In early season, coefficients of the relationship between elevation and phloem feeders were generally positive on the western side of the field with values ranging from 0.06 to 0.17, and negative at the northeastern edge and southeastern area of the field with values ranging from -0.02 to -0.43 (see S1 Fig for elevation map). The positive relationships corresponded to the higher elevations on the western than the eastern side of the field (Fig 5). By mid-season, the negative relationship expanded to the central, southeastern, and northwestern regions with values ranging from -0.021 to -0.16, but, by late season coefficients shifted to being largely positive ranging from 0.086 to 1.3. This shift is especially notable at sites 47 and 49 at the northeastern edge of the field where coefficient values over the season change from -0.43 to 1.30 and corresponds to sites at the lowest elevations in the field. Credence in coefficient values decreased over time, with standard errors increasing in many of the values, especially in the western side of the field.

#### Slope (SLOPE)

**Fig 6:**
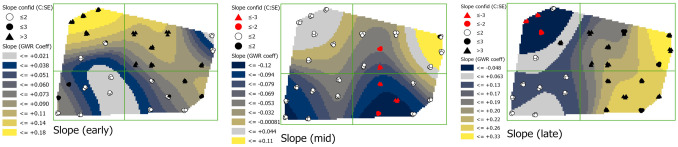
Maps showing early, mid-, and late season SLOPE coefficients with estimations of credence in coefficient values. Coefficient surface values were symbolized using a geometric interval classification scheme from blue to gold, with grey indicating the interval including zero (if present).

In early season, coefficients of the relationship between slope and phloem feeders were generally positive throughout the field with values ranging from 0.073 to 0.180 (Fig 6; see S2 Fig for slope map). In mid-season, there was a negative relationship between slope and phloem-feeders in the southeastern region with values ranging from -0.079 to -0.12, but, by late season coefficients shifted to being largely positive again with values ranging from 0.063 to 0.33. This shift is especially notable at sites 4 and 6 where coefficient values change from 0.18 to– 0.48 and sites 48, 34, 36, and 40 where coefficient values change from -0.079 to 0.280. Credence in coefficient values increased over time, with standard errors decreasing in many of the values, especially late season.

#### Southward facing aspect (SI)

**Fig 7:**
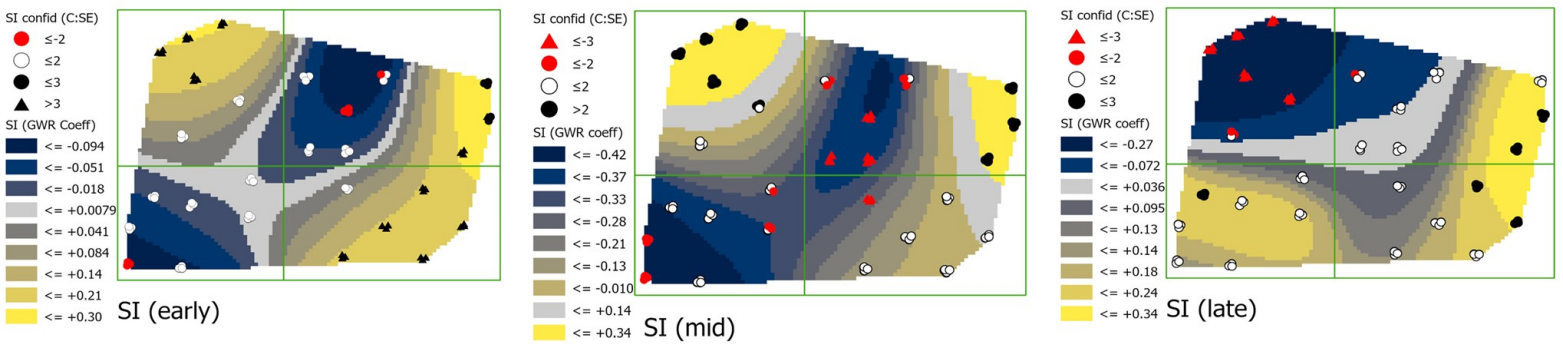
Maps showing early, mid-, and late season southward facing aspect (SI) coefficients with estimations of credence in coefficient values. Coefficient surface values were symbolized using a geometric interval classification scheme from blue to gold, with grey indicating the interval including zero (if present).

In early season, coefficients of the relationship between SI and phloem feeders were generally positive in the eastern side and the northwestern corner of the field with values ranging from 0.21 to 0.30 (Fig 7; see S3 Fig for SI map). By mid-season, the positive relationship decreased in area and was found only in the northeastern and northwestern corners with a value of 0.34. A negative relationship occurred in the central region with values ranging from -0.33 to -0.42. By late season, coefficients in the northwestern corner shifted and was especially notable at sites 3, 4, 6 and 8 in the northwestern corner of the field where coefficient values change from 0.30 to -0.27. Credence in coefficient values decreased over time, with standard errors increasing in many of the values, especially in the southern area of the field.

#### Percent silt (SILTNR)

**Fig 8:**
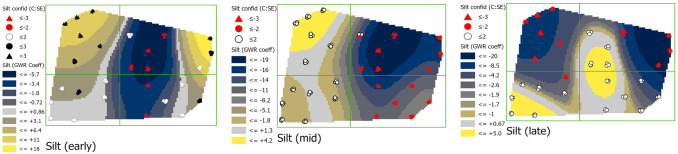
Maps showing early, mid-, and late season percent of silt without rock content (SILTNR) coefficients with estimations of credence in coefficient values. Coefficient surface values were symbolized using a geometric interval classification scheme from blue to gold, with grey indicating the interval including zero (if present).

In the early season, coefficients of the relationship between the proportion of silt in the soil at a 0–15 cm depth and phloem feeders were positive in the northwestern and northeastern corners, and negative in the central area of the field (Fig 8; see S6A Fig for SILTNR map). The positive relationship corresponded with soils having the lowest proportion of silt (0.178–0.299) and the negative relationship corresponded to soils having a higher proportion of silt (0.315–0.355). By mid-season, the negative relationship increased across the eastern region, and even shifted direction in the northeastern corner. By late season coefficients also shifted to being largely negative in the northwestern corner. The shifts in coefficients are especially notable at sites 3, 4, 6, 8, 47 and 49 and correspond to values changing from 18.0 to -20.0. Over the entire field, credence in coefficient values decreased over time, with standard errors increasing especially in the central and southern regions.

#### NDVI

**Fig 9:**
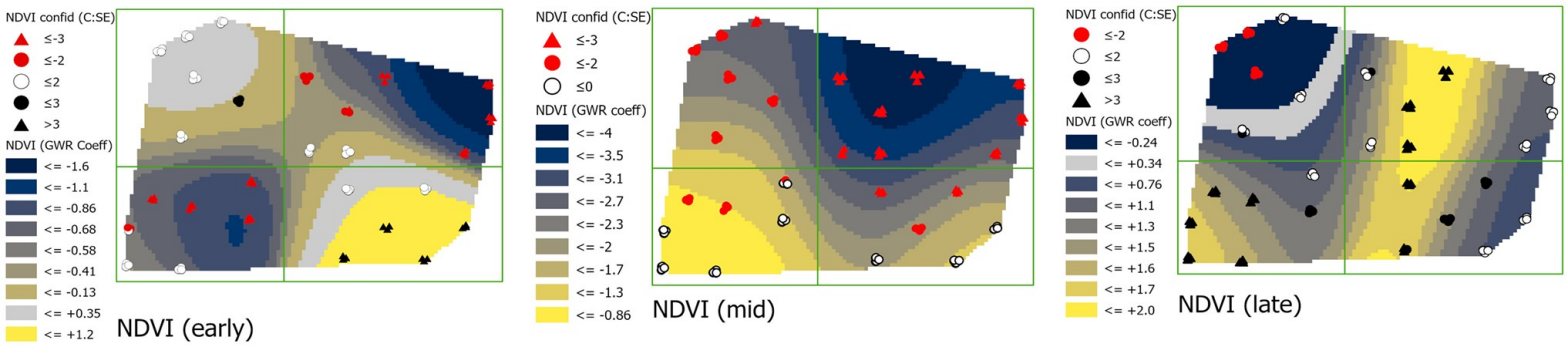
Maps showing early, mid-, and late season NDVI coefficients with estimations of credence in coefficient values. Coefficient surface values were symbolized using a geometric interval classification scheme from blue to gold, with grey indicating the interval including zero (if present).

In early season, coefficients of the relationship between NDVI and phloem feeders were generally positive in the southeastern corner of the field with a value of 1.2, and negative in the northeastern and southwestern corners, with values ranging from -0.86 to -1.2 (Fig 9; see S4 Fig for NDVI maps). By mid-season, the negative relationship expanded across the entire northern region, with values ranging from -2.3 to -4.0. But, by late season, coefficients shifted to being largely positive in all but the northwestern corner with values ranging from 1.1 to 2.0. This shift is especially notable at sites 30, 32, 34 and 36 in the central region where coefficient values change from -4.0 to 2.0. Credence in coefficient values increased over time, with standard errors decreasing in many of the values, especially in the southern and northeastern areas of the field.

#### Natural enemies (INS_NET_1)

**Fig 10:**
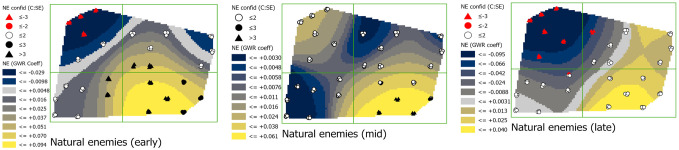
Maps showing early, mid-, and late season natural enemies (INS_NET_1) coefficients with estimations of credence in coefficient values. Coefficient surface values were symbolized using a geometric interval classification scheme from blue to gold, with grey indicating the interval including zero (if present).

In early season, coefficients of the relationship between natural enemy abundance and phloem feeders were negative in the northwestern corner (-0.029) but the relationship was predominantly positive in central and southeastern regions, ranging from 0.051 to 0.094 (Fig 10). By mid-season, the positive relationship increased across the entire field, but credence in the coefficients was low in all but the southeastern corner of the field, where coefficients were 0.038 to 0.061. Coefficients were again negative late season in the northwestern corner of the field with values ranging from -0.066 to -0.095. Credence in coefficient values decreased over time, with standard errors increasing in many of the values, especially in the southern and northeastern areas of the field.

#### Spiders (SPI_TOT_1)

**Fig 11:**
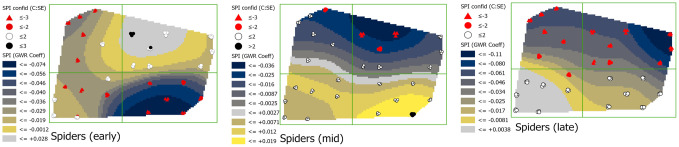
Maps showing early, mid-, and late season spiders (SPI_TOT_1) coefficients with estimations of credence in coefficient values. Coefficient surface values were symbolized using a geometric interval classification scheme from blue to gold, with grey indicating the interval including zero (if present).

In early season, coefficients of the relationship between spiders and phloem feeders were generally negative in the southern and western areas of the field with coefficients ranging from -0.029 to -0.074 (Fig 11). By mid-season, this shifted to a negative relationship only at the northernmost edge where coefficients ranged from -0.025 to -0.036, and by late season coefficients were largely negative across the entire northern region with coefficients ranging from -0.036 to -0.11. Credence in coefficient values was higher for the negative coefficient values, and where coefficients shifted from negative to positive in mid-season, credence in those values declined. However, credence in coefficient values was consistently highest in the northern region of the field.

## Discussion

The exploratory regression and review of non-spatial models, both global and seasonally grouped, yielded a few clues about the overall responses of phloem feeders to environmental variables. The parameters described in the hypothesized model (i.e. Model 5) fit well with what is known of phloem-feeding insects’ drivers that determine their abundance and distribution. However, other environmental variables, especially related to weather conditions, were shown to provide a better fitting global model, i.e. when there was no seasonal or spatial differentiation. Of the weather variables, wind speed had the greatest influence on phloem feeder abundance, with a positive correlation. However, there likely is an interaction between wind speed and elevation and our global OLS model did indicate multicollinearity. Once the data were broken out into seasonal values, we lost the ability to evaluate wind speed as a factor in the distribution of phloem feeders, so this result is inconclusive. We would need to have a much higher density of temporal sampling in conjunction with the spatial sampling to measure this. Kumar and Kumar [62] found a negative relationship between aphids and wind speed in cowpea, but the wind speeds reported (0.81 mph to 3.78 mph) were lower than wind speeds in this present study area (1.60 mph to 4.13 mph). Additionally, a study in the Columbia Basin [63] found no association between wind speed and aphids in potato.

With the responses grouped by season, and without weather variables, plant health was a significant predictor of phloem feeder abundance in the non-spatial models. Plant health as measured by NDVI can indicate both an attractant to phloem feeders and the effects of herbivory. Lower NDVI values are indicative of plant stress from, for example, nutrient and water deficiencies, and injury [48–52]. In this study, the strong negative relationship between NDVI and phloem feeders indicated that they were more abundant where NDVI values were lower. This negative correlation observed suggests that phloem feeders preferred weaker *M*. *giganteus* plants and/or are stressing plants through their herbivory. Finally, spiders and, less so natural enemies, showed a significant negative relationship with phloem feeders, indicating lower abundance of phloem feeders where numbers of natural enemies and spiders were higher during early and late seasons.

The GWR models provided spatially explicit information about how phloem feeders responded in complex ways to environmental variability. But GWR results can be difficult to interpret when relationships among multiple variables are continually shifting in space and over time. In this study, shifting effects were the norm across all seven of the variables (Fig 6), as the correlation with phloem feeder abundance of most variables shifted between negative and positive from one region of the field to another. Variables with the greatest spatial consistency in their effects included: spiders, which were predominantly negative at all times; NDVI, which was negative across the entire field in mid-season; and natural enemies, which was positive across most of the field in early season and the entire field in mid-season. The correlations between phloem feeders and environmental variables also shifted in time, although there were a few exceptions among sites. Temporally, more consistent effects in all variables were seen in the eastern region of the field over the course of the study period, especially the northeastern corner, where phloem feeder responses to slope and southward facing aspect were consistently positive. This northeastern corner of the site is low lying, very flat and protected by trees (S1, S2 and S5 Figs).

Both spiders and insect natural enemies, had negative associations with phloem feeders, but mostly in the late season for natural enemies. These predator species are generalists and are well known to be effective against phloem feeders [64–68] and may be exerting some control of phloem feeders in *M*. *giganteus*. The magnitude of the coefficients was relatively low for these predators, but they are omnivores and often feed on alternative prey, seeds, or other food items. Molecular gut content analysis of the spiders (JMS, unpublished data) in this study indicated frequent positives for the DNA of aphids and thrips, but also dipteran species as alternative prey that were highly prevalent over all sites in the field, especially early and mid-season.

Soil and terrain variables were also included in the GWR models, and the strength of their effects was especially evident in early season models when credence in the coefficient values was generally high. Here, elevation, slope and the southward facing aspect were, especially in early and late seasons, positively correlated with phloem feeder abundance. The positive effects of elevation in GWR models stands in contrast to the strong negative effects of elevation described in the non-spatial global and seasonal models. In addition, as stated above, elevation and wind speed may interact in regard to phloem feeder distribution, so this result is also inconclusive.

The only study of phloem feeders’ response to elevation over time [11] found that phloem feeders on striped maple trees did not respond to changes in elevation until later in the season when their abundance at lower elevations increased exponentially, likely because of warmer temperatures found at lower elevations. The elevation ranges of their study sites were much higher (980 to 1570 mamsl) than those found here (98 to 109 mamsl). There are a few studies of phloem feeders’ response to changes in topographic and edaphic factors within fields. However, these studies report responses to the average of phloem feeder abundance over sampling dates within a season, and they did this because topographic and edaphic factors would not change over time. These include studies in cereal and wheat crops where no relationship was found in the average responses of aphids to elevation and aspect [7, 12, 69] a positive relationship was found in the average aphid responses to slope [69] and a negative relationship was found in the average aphid response to the proportion of silt in the soil [69]. In the present study, phloem feeders similarly responded to the proportion of silt in the soil; the analysis shows that phloem feeder abundance decreased as the proportion of silt in the soil increased.

Precision agriculture (PA) can increase efficiency in the use of pesticides and reduce pesticide contamination. But effective use of PA requires a clear understanding of field-scale ecological relationships that change over space and time. Understanding the mechanism(s) underlying the spatial and temporal distribution of arthropod pests relative to environmental factors and predatory arthropod abundance allows us to identify areas where, for example, the use of select insecticides that are less harmful to non-target species could be applied and habitat management schemes could be incorporated to conserve and enhance the diversity and abundance of beneficial arthropods with the field. But, unravelling the complex and changing relationships among environmental variables requires fine scale local data, and the use of analytical methods, like geographically weighted regression, that are spatially explicit. Applications of these results could include the identification of management zones where models of phloem feeder responses to the environment are consistent in space and time, hence informing PA applications. This kind of information is essential to building better PA tools that can inform producers about the specific issues evident within their agricultural fields and enhancing their ability to farm sustainably.

## Supporting information

S1 Fig Map of elevation contours and insect sample points. (PDF)

S2 Fig Map of percent slope (SLOPE). (PDF)

S3 Fig Maps of aspect variables. A. Southward facing aspect (SI). B. Westward facing aspect (WI).
(PDF)

S4 Fig Maps of normalized difference of vegetation index (NDVI) for 2015 and 2016. A. Map of NDVI, 6 July 2015. B. Map of NDVI, 3 August 2015. C. Map of NDVI, 31 August 2015. D. Map of NDVI, 6 July 2016. E. Map of NDVI, 3 August 2016. F. Map of NDVI, 7 September 2016.
(PDF)

S5 Fig Map of distance to the nearest woodland edge (DISTFOR). (PDF)

S6 Fig Description of soil sampling, particle size analysis, and results of kriging interpolations. A. Map showing kriged surface of proportion of silt without gravel fraction (SILTNR). B. Map showing kriged surface of proportion of sand without gravel fraction (SANDNR). C. Map showing kriged surface of proportion of clay without gravel fraction (CLAYNR). D. Map showing kriged surface of proportion of gravel (GRAVEL). E. Map showing kriged surface of proportion of silt including gravel fraction (SILT). F. Map showing kriged surface of proportion of sand including gravel fraction (SAND). G. Map showing kriged surface of proportion of clay including gravel fraction (CLAY).
(DOCX)

S7 Fig Coefficient values for selected sample locations. SiteID refers to the main sample ID, shown in Fig 1. Rep (1, 2, or 3), refers to the three sample points located around each main point. Colors indicate the value of credence, or the ratio of the coefficient and standard error. No color indicates no or little credence. Light color indicates moderate levels of credence. Bold color indicates high levels of credence.
(PDF)
