# Supplementary figures and images for: Responses to environmental variability by herbivorous insects and their natural enemies within a bioenergy crop, Miscanthus x giganteus
Source: PLoS One. 2021 Feb 16;16(2):e0246855. doi: 10.1371/journal.pone.0246855 (PMC7886118; doi:10.1371/journal.pone.0246855)

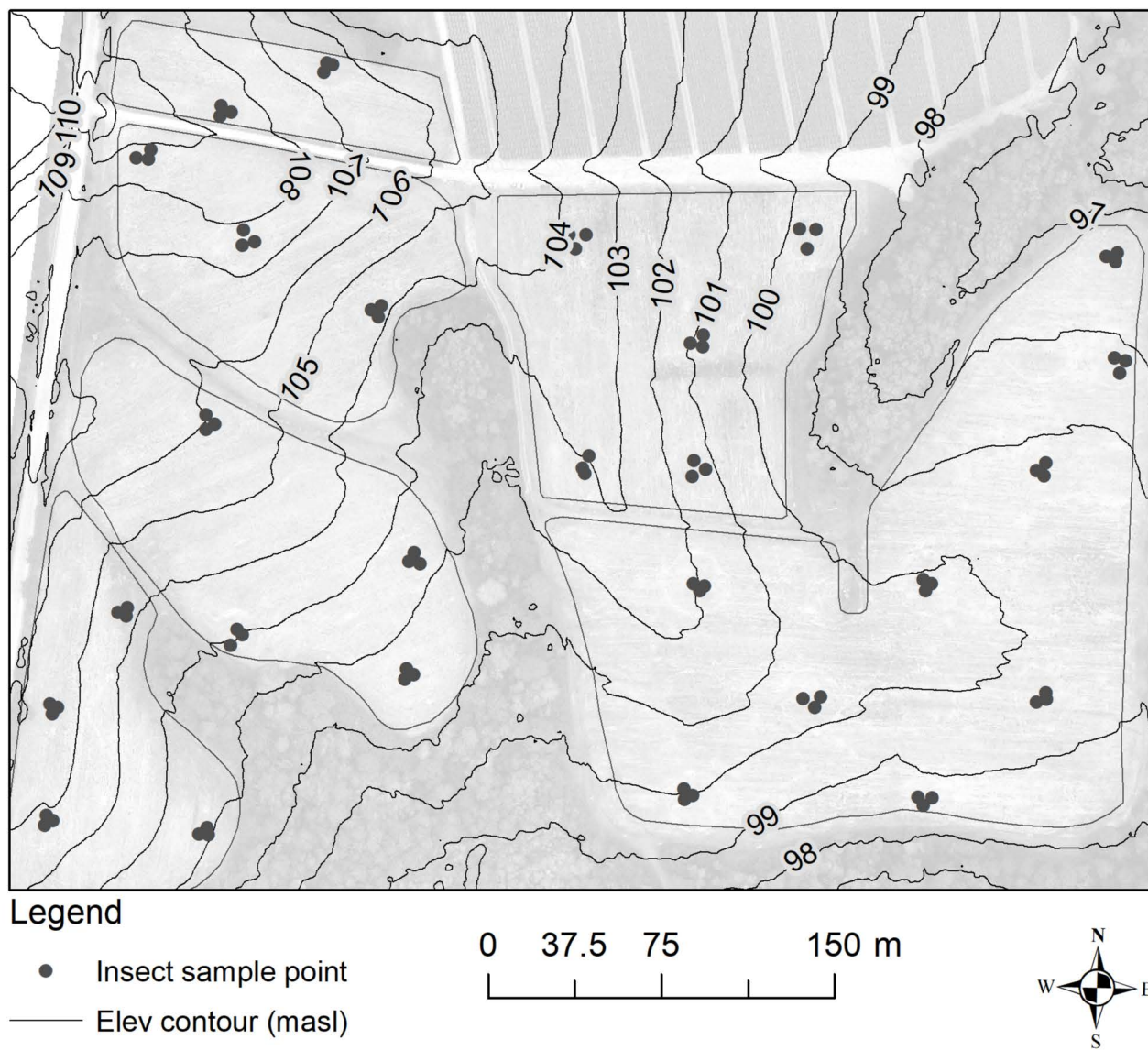

S1 Figure. Topographic contours of study area with insect sample points.

Supplement: S1 Fig — (PDF) [file pone.0246855.s001.pdf]
