# Supplementary material for: Responses to environmental variability by herbivorous insects and their natural enemies within a bioenergy crop, Miscanthus x giganteus
Source: PLoS One. 2021 Feb 16;16(2):e0246855. doi: 10.1371/journal.pone.0246855 (PMC7886118; doi:10.1371/journal.pone.0246855)
Supplement: S2 Fig — (PDF) [file pone.0246855.s002.pdf]

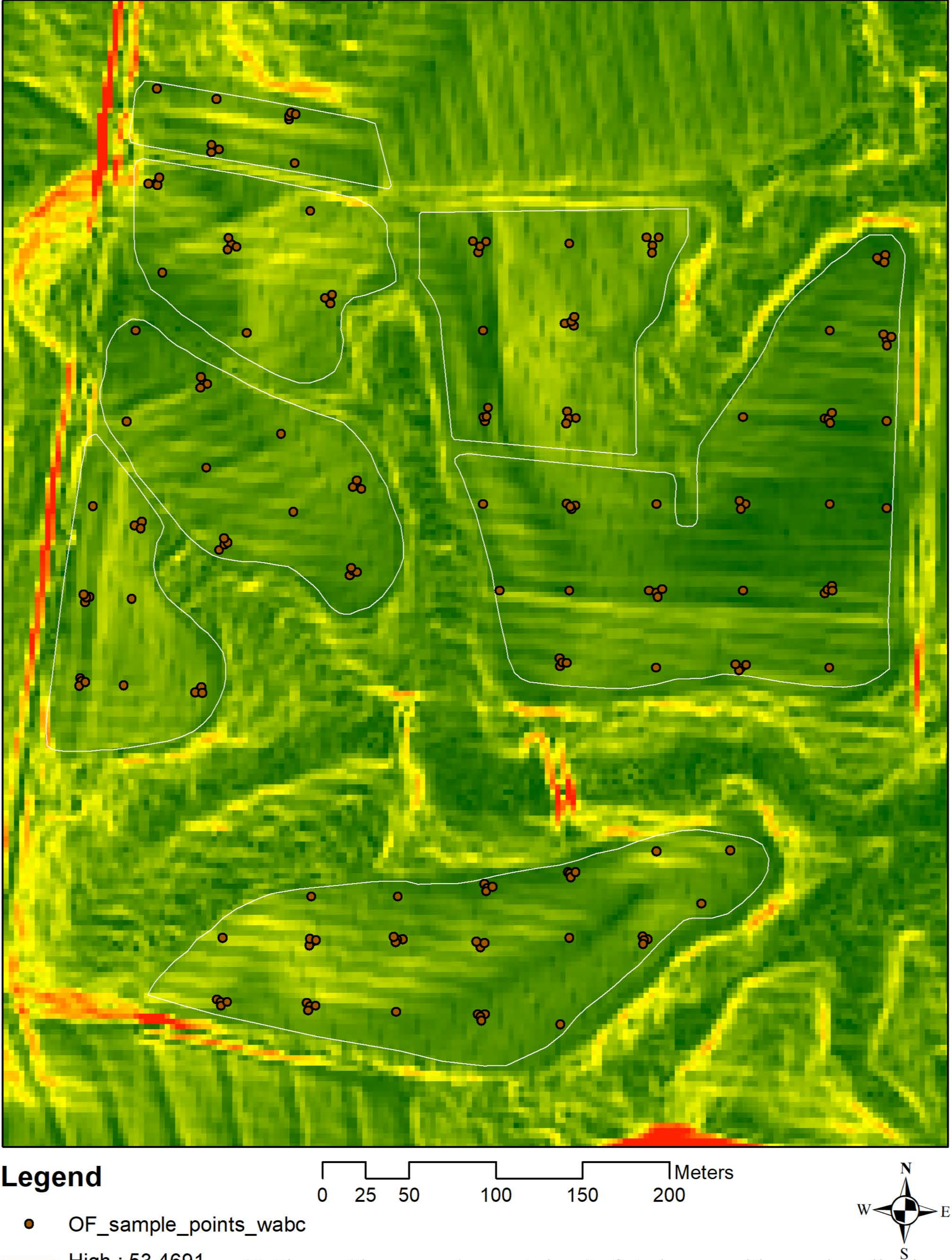

S2 Figure. Slope map (percent slope) of study area with sample collection points. An additional field south of the study area appears in this map, but was not included in the analysis.
