# Supplementary material for: Responses to environmental variability by herbivorous insects and their natural enemies within a bioenergy crop, Miscanthus x giganteus
Source: PLoS One. 2021 Feb 16;16(2):e0246855. doi: 10.1371/journal.pone.0246855 (PMC7886118; doi:10.1371/journal.pone.0246855)
Supplement: S3 Fig — A. Southward facing aspect (SI). B. Westward facing aspect (WI). (PDF) [file pone.0246855.s003.pdf]

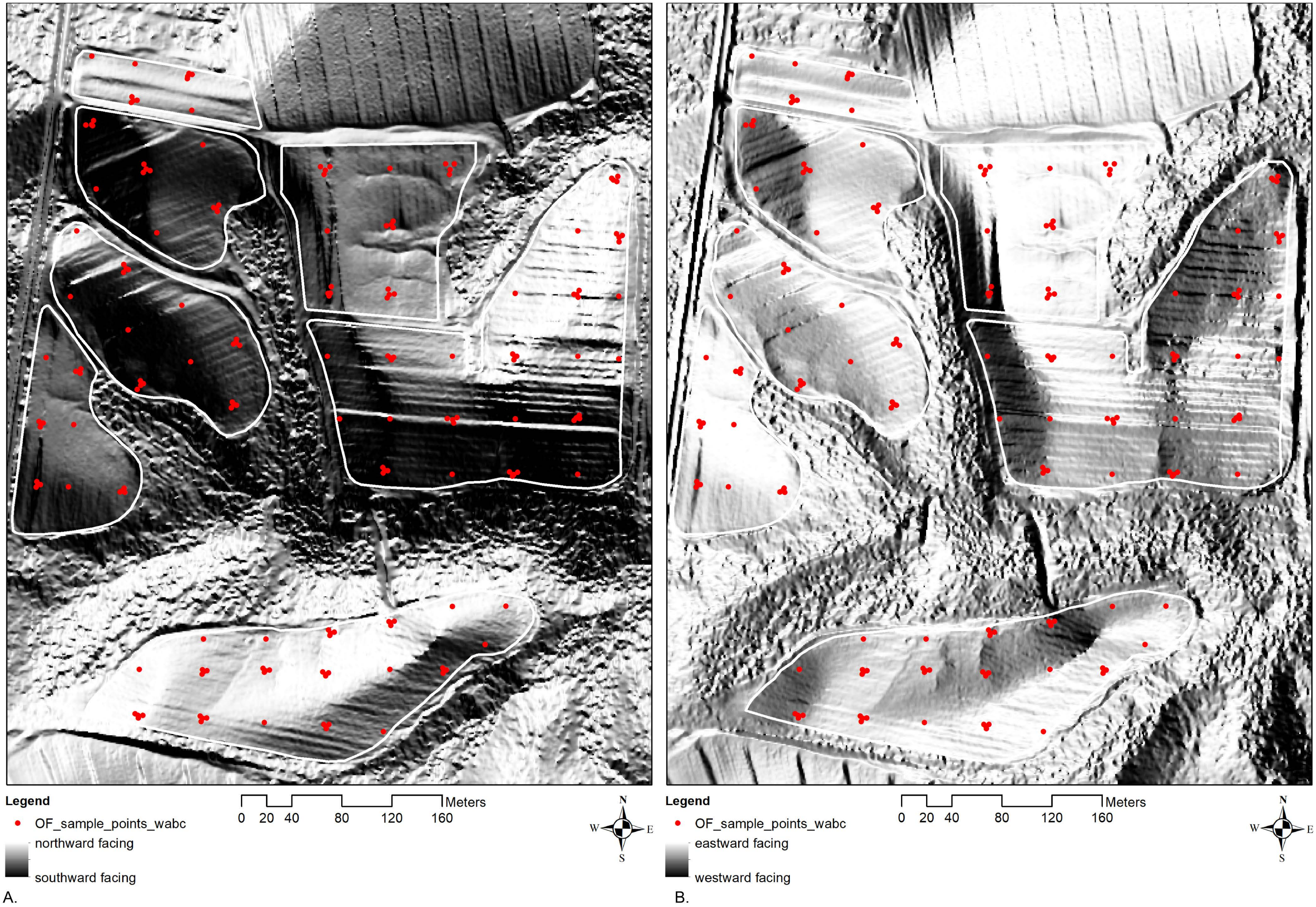

S3 Figure. Maps showing the two axial components of aspect including southward index (SI) and westward index (WI) along with insect sample points. An additional field south of the study area appears in this map, but was not included in the analysis. A. SI = cos(wind direction, radians), [-1, 1]; southward to northward facing; B. WI = sin(wind direction, radians), [-1, 1]; westward to eastward facing.
