# Supplementary material for: Responses to environmental variability by herbivorous insects and their natural enemies within a bioenergy crop, Miscanthus x giganteus
Source: PLoS One. 2021 Feb 16;16(2):e0246855. doi: 10.1371/journal.pone.0246855 (PMC7886118; doi:10.1371/journal.pone.0246855)
Supplement: S4 Fig — A. Map of NDVI, 6 July 2015. B. Map of NDVI, 3 August 2015. C. Map of NDVI, 31 August 2015. D. Map of NDVI, 6 July 2016. E. Map of NDVI, 3 August 2016. F. Map of NDVI, 7 September 2016. (PDF) [file pone.0246855.s004.pdf]

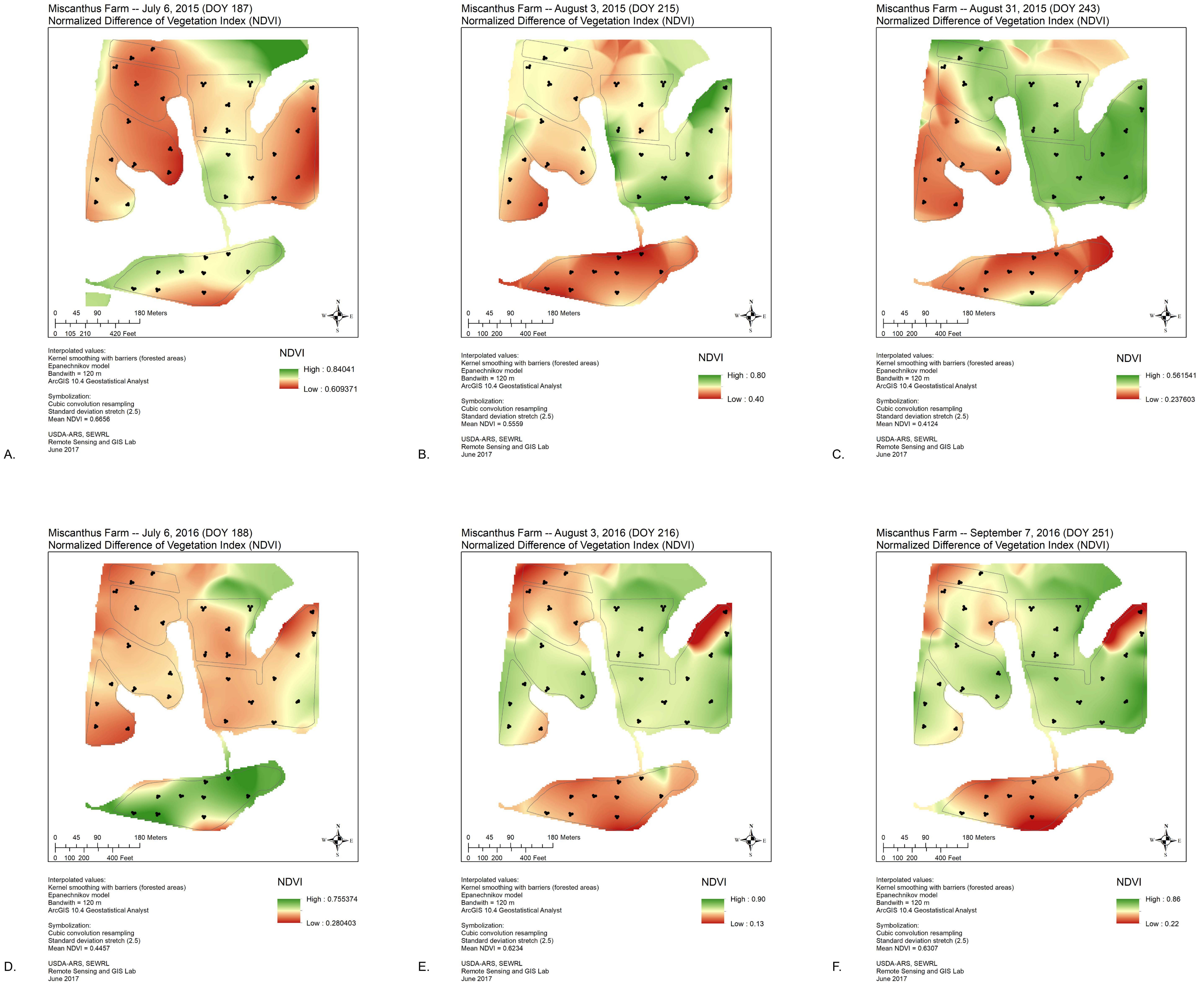

S4 Figure. Maps of Normalized Difference of Vegetation Index (NDVI), interpolated from point values. Also show are insect sample points. An additional field south of the study area appears in this map, but was not included in the analysis. A. NDVI for July 6, 2015; B. NDVI for August 3, 2015; C. NDVI for August 31, 2015; D. NDVI for July 6, 2016; E. NDVI for August 3, 2016; F. NDVI for September 7, 2016.
