# Supplementary material for: Responses to environmental variability by herbivorous insects and their natural enemies within a bioenergy crop, Miscanthus x giganteus
Source: PLoS One. 2021 Feb 16;16(2):e0246855. doi: 10.1371/journal.pone.0246855 (PMC7886118; doi:10.1371/journal.pone.0246855)
Supplement: S5 Fig — (PDF) [file pone.0246855.s005.pdf]

Distance from forest edge

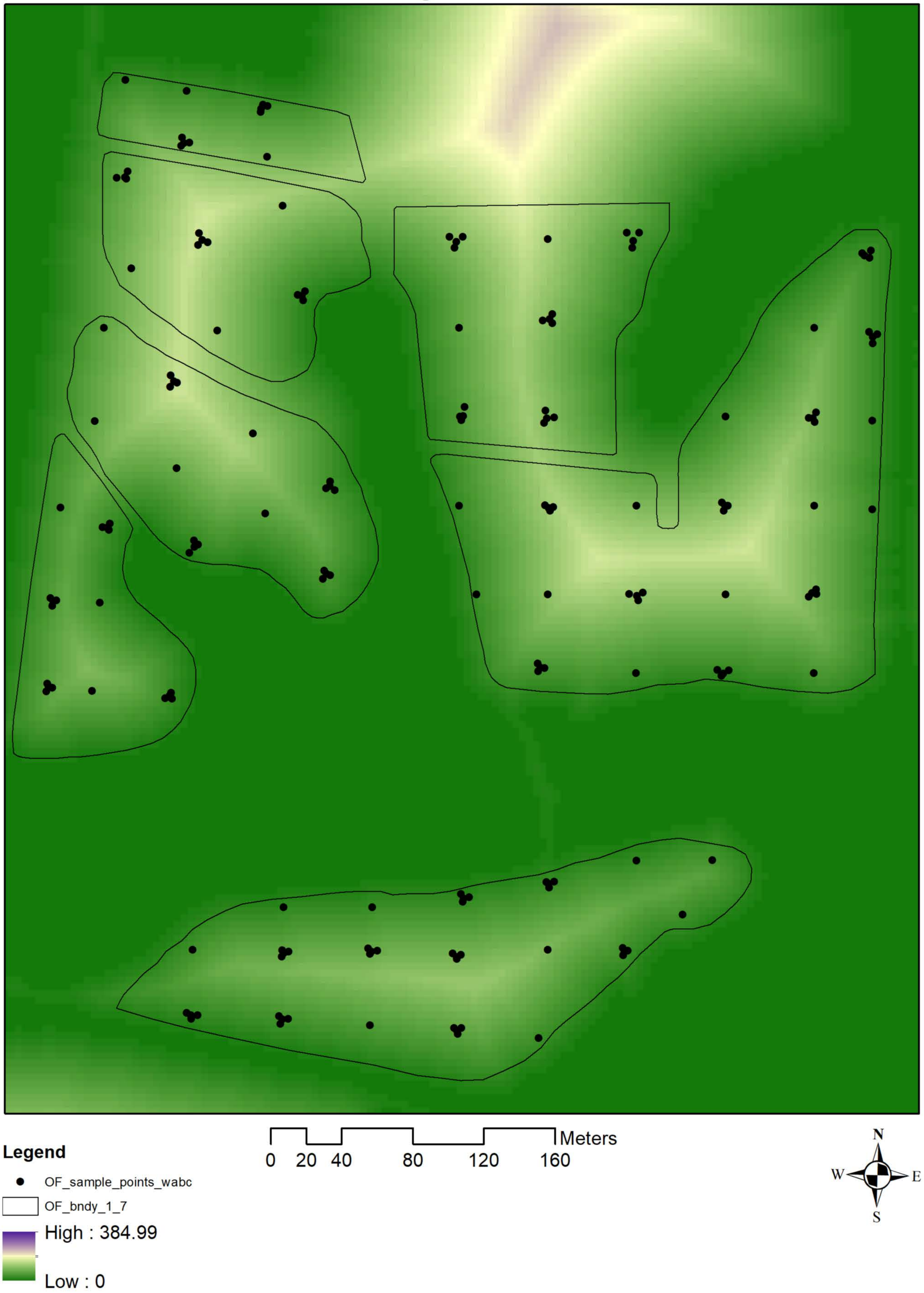

S5 Figure. Map showing distance to edge of forest (meters) with insect and soil core sample points. An additional field south of the study area appears in this map, but was not included in the analysis.
