# Supplementary material for: Responses to environmental variability by herbivorous insects and their natural enemies within a bioenergy crop, Miscanthus x giganteus
Source: PLoS One. 2021 Feb 16;16(2):e0246855. doi: 10.1371/journal.pone.0246855 (PMC7886118; doi:10.1371/journal.pone.0246855)
Supplement: S6 Fig — A. Map showing kriged surface of proportion of silt without gravel fraction (SILTNR). B. Map showing kriged surface of proportion of sand without gravel fraction (SANDNR). C. Map showing kriged surface of proportion of clay without gravel fraction (CLAYNR). D. Map showing kriged surface of proportion of gravel (GRAVEL). E. Map showing kriged surface of proportion of silt including gravel fraction (SILT). F. Map showing kriged surface of proportion of sand including gravel fraction (SAND). G. Map showing kriged surface of proportion of clay including gravel fraction (CLAY). (DOCX) [file pone.0246855.s006.docx]

**Soil Sampling and Particle Size Analysis**

Soil cores were taken from each of 73 sampling sites on 12 December 2016 using a tractor mounted Giddings probe (Giddings Machine Company, Windsor, CO) fitted with a clear plastic sleeve inside the sampling tube.  Each core was plugged with paper towels to minimize disturbance of the soil surface layer, capped, transported to the lab, and stored at 4^o^C until processed. Cores were sub-sampled by depth increment (0-15, 15-30, 30-45, and 45-60cm). Depth increment samples were air-dried 48 hours followed by the disruption of clods with a wooden rolling pin. Roots were removed by hand, and the remaining sample was sieved (#10; < 2mm). Material above the sieve was dried 24 hours at 105 °C and the mass recorded as stones. Three sub-samples (~10g) of material passing the sieve were taken to determine an average soil oven dry mass and water content as above.

Fifty grams (25g if high clay) of the < 2mm air dried soil from the 0-15cm depth increment was shaken for 15 minutes at 125 rpm in 100ml of a 5% hexametaphosphate solution and quantitatively transferred onto a #18 sieve (1mm) stacked onto a #45 sieve (0.355mm) and a pre-weighed pan. Samples were sequentially washed through each sieve using a squirt bottle with deionized water. Each sieve was inverted and material above the sieve washed into a collection pan and then into a pre-weighed 400 ml beaker. Samples from the #18 and #45 sieved were dried for 48 hours at 105 ^o^ C and mass recorded for particle size ranges 1-2mm and 0.350-1mm respectively. The pan under the sieves was weighed and the soil and water were quantitatively transferred into a 400ml beaker. While the material in the 400ml beaker was uniformly suspended in solution using a magnetic stir bar, three 20ml aliquots were transferred into separate pre-weighed 50ml beakers, oven dried for 24 hours and weighed to determine average mass per ml. The average mass per ml was multiplied by the mass of the soil + water in the pre-weighed pan to calculate the soil mass < 0.350mm. Two additional 20ml aliquots were transferred into 100ml beakers, made up to an 80ml volume with deionized water, and analyzed on a Micromeritics Saturn Digitizer II high definition digital particle size analyzer (Micromeritics Instrument Corporation, Norcross, GA, U.S.A.) using deionized water as the suspending solution and programmed to report the %mass of fractions < 0.002mm, 0.002-0.050mm, 0.050-0.100mm, and > 0.250mm nominal particle diameter. Resulting percentages were multiplied by the soil mass < 0.35 mm and the mass of material > 0.050 mm was added to the sand fraction to report clay (< 0.002 mm), silt (0.002-0.040 mm), sand (0.050-2.0 mm), stone (> 2 mm) content.

**Soil mapping**

Values for soil proportions were mapped using Esri® ArcMap (version 10.7.1, advanced). Using kriging tools in the Geostatistical Analyst extension, data were interpolated to create maps of each soil fraction. Ordinary kriging was used and throughout each interpolation process, settings were selected to optimize the model fit with a smoothing factor of 0.2 and output raster datasets had a pixel resolution of 2m. Resulting datasets provide a continuous surface of soil proportion values (Figures A through G). Values at the sample locations were extracted from the surface rasters and these were the values used for subsequent regression analysis.


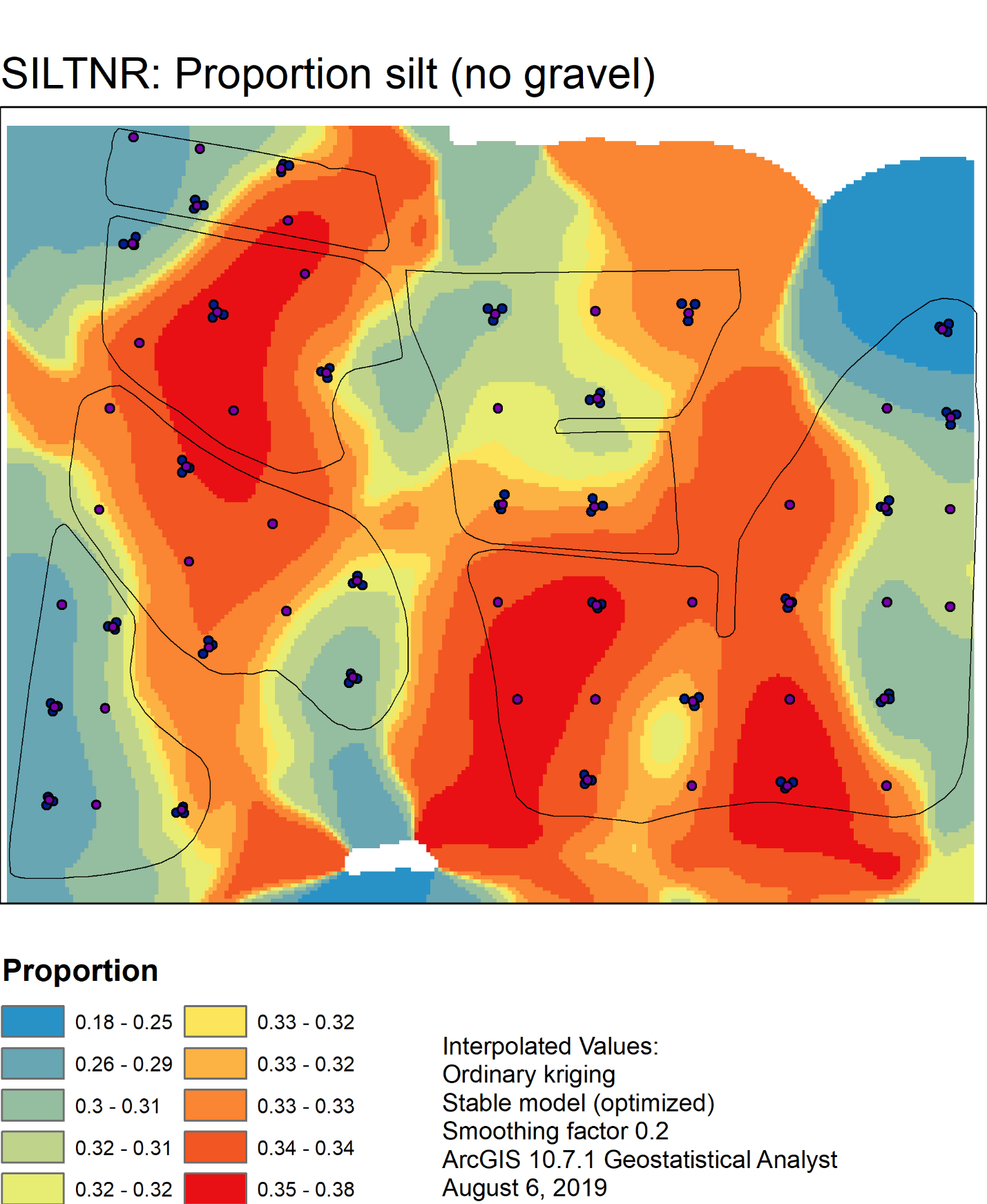


**S6A Fig. Map showing kriged surface of proportion of silt without gravel fraction (SILTNR).**


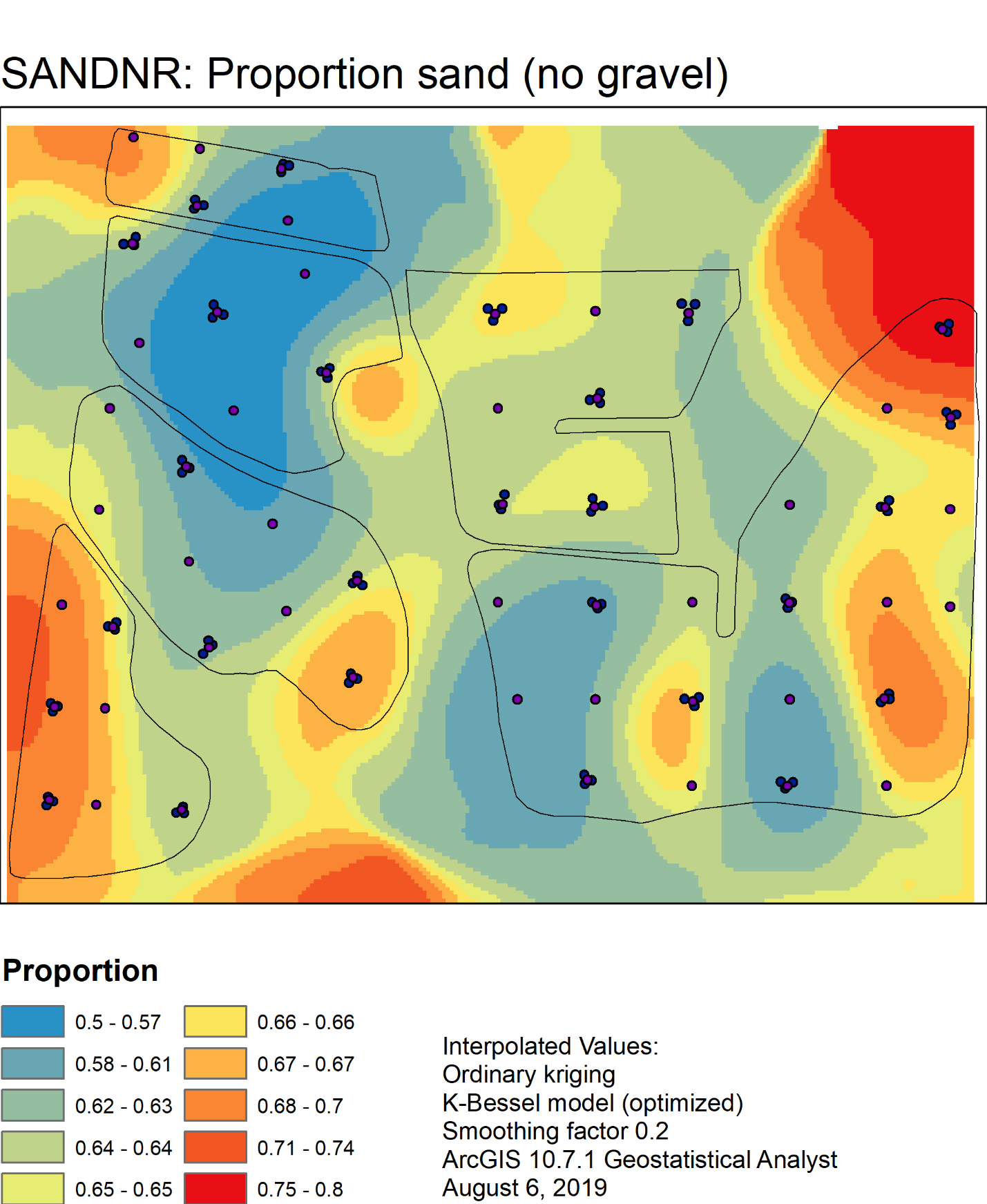


**S6B Fig. Map showing kriged surface of proportion of sand without gravel fraction (SANDNR).**


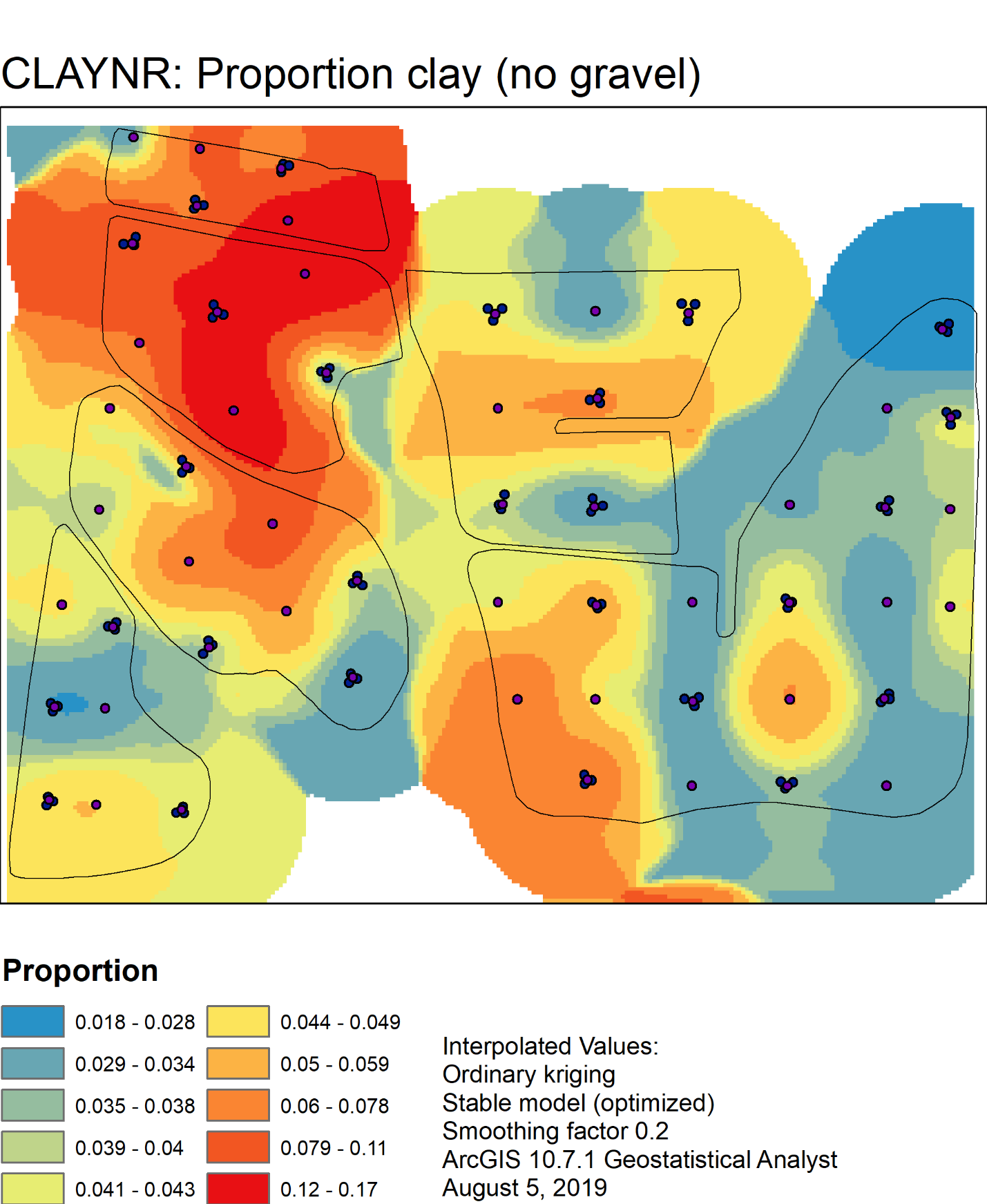


**S6C Fig. Map showing kriged surface of proportion of clay without gravel fraction (CLAYNR).**


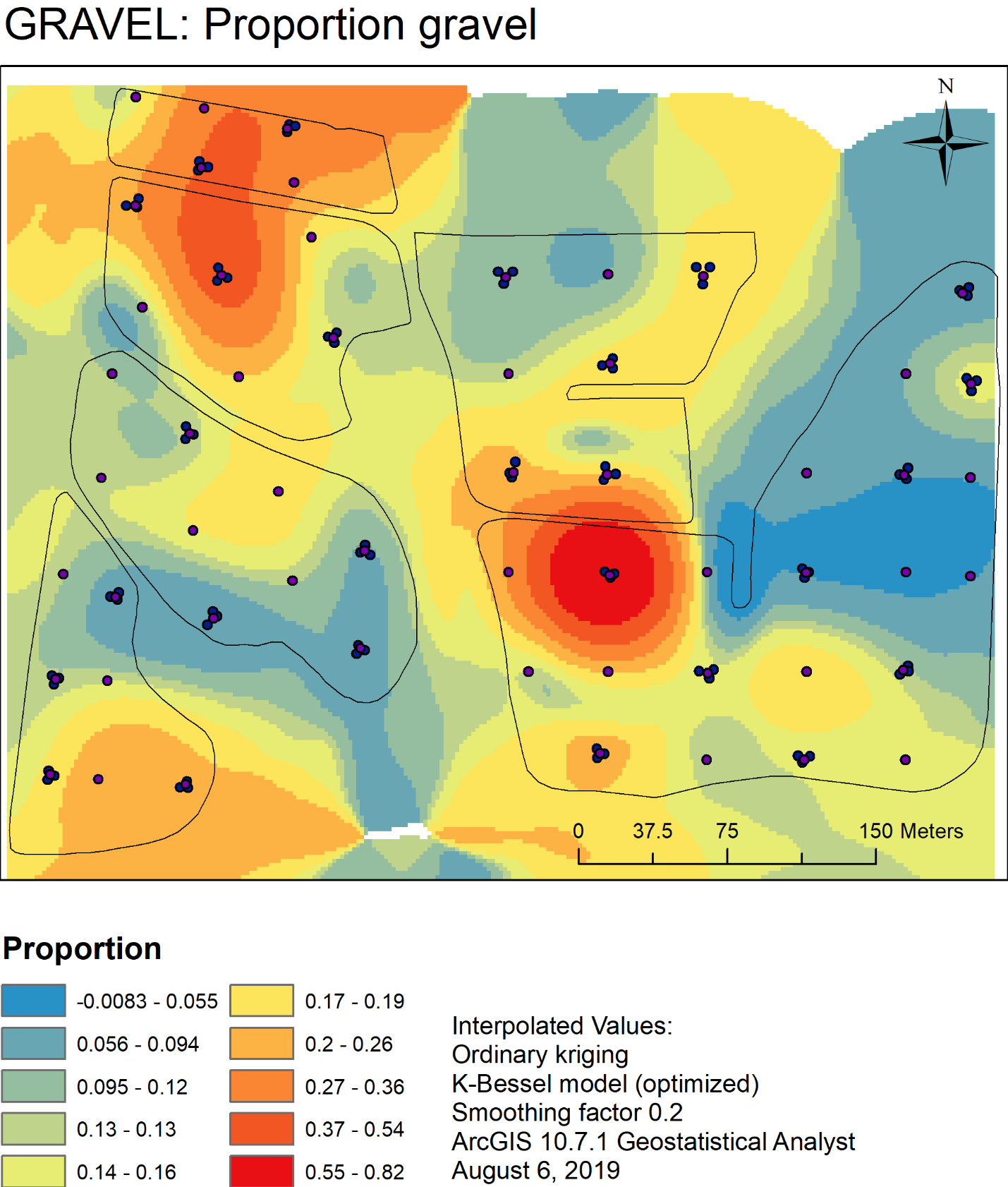


**S6D Fig. Map showing kriged surface of proportion of gravel (GRAVEL).**


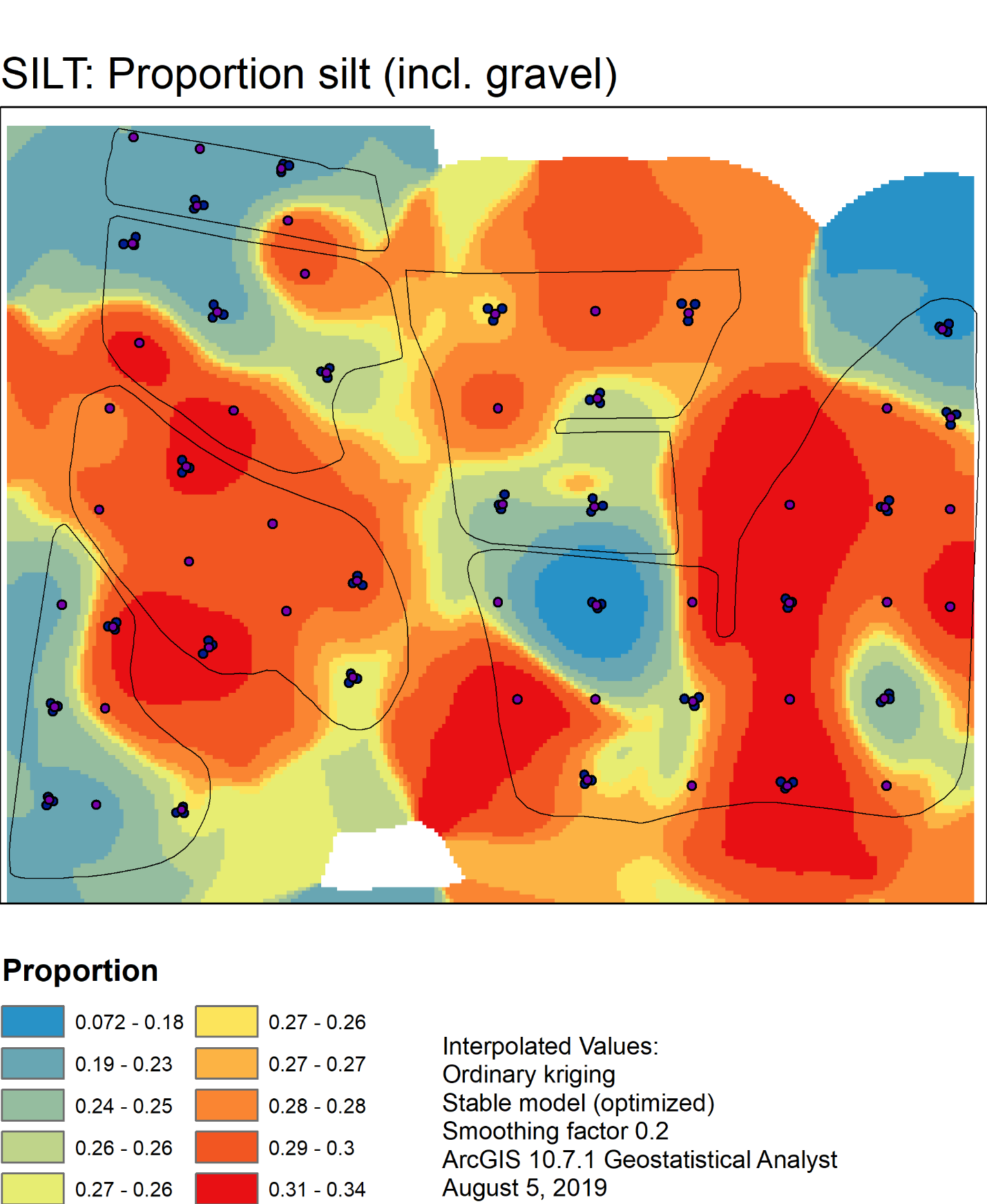


**S6E Fig. Map showing kriged surface of proportion of silt including gravel fraction (SILT).**


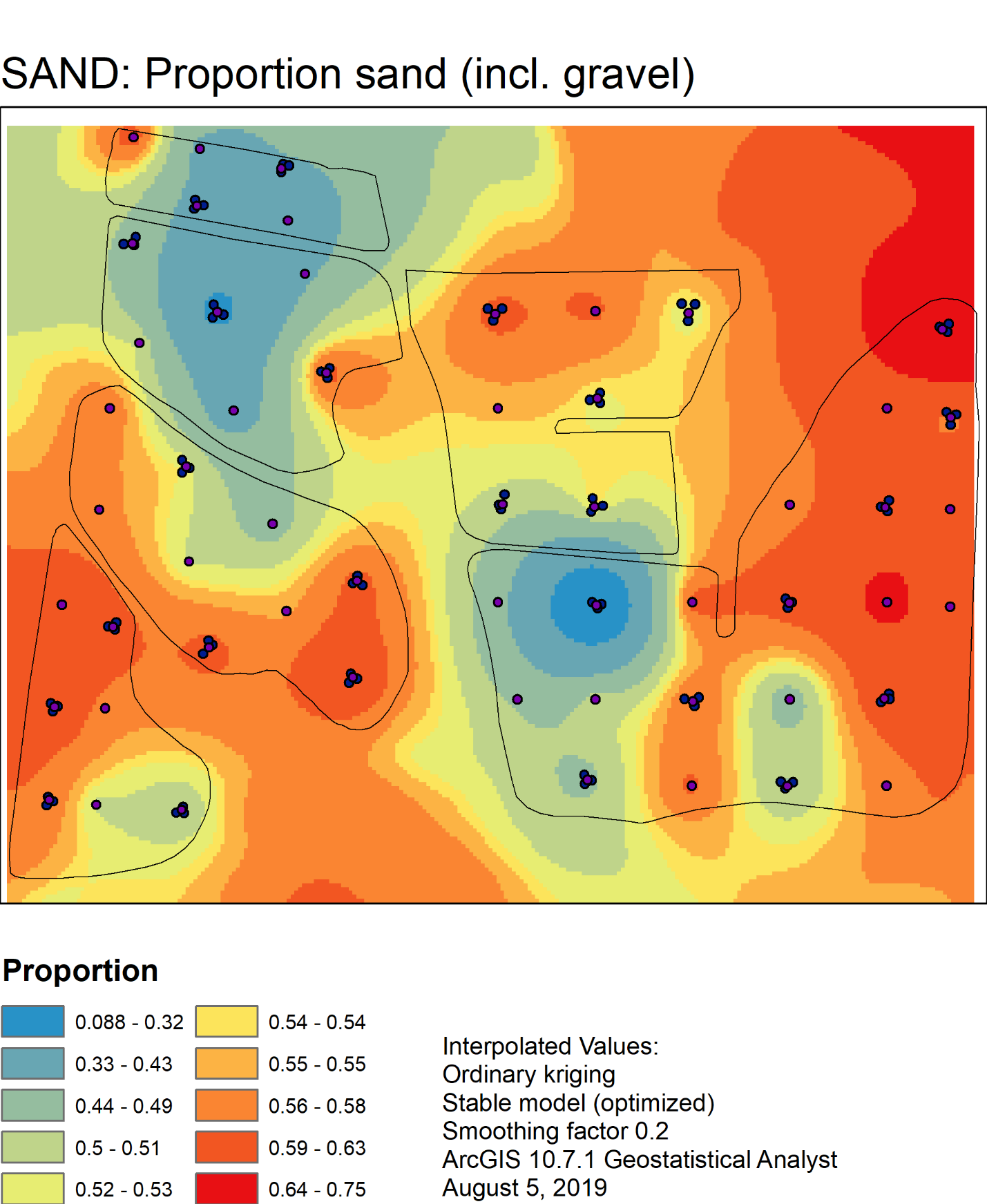


**S6F Fig. Map showing kriged surface of proportion of sand including gravel fraction (SAND).**


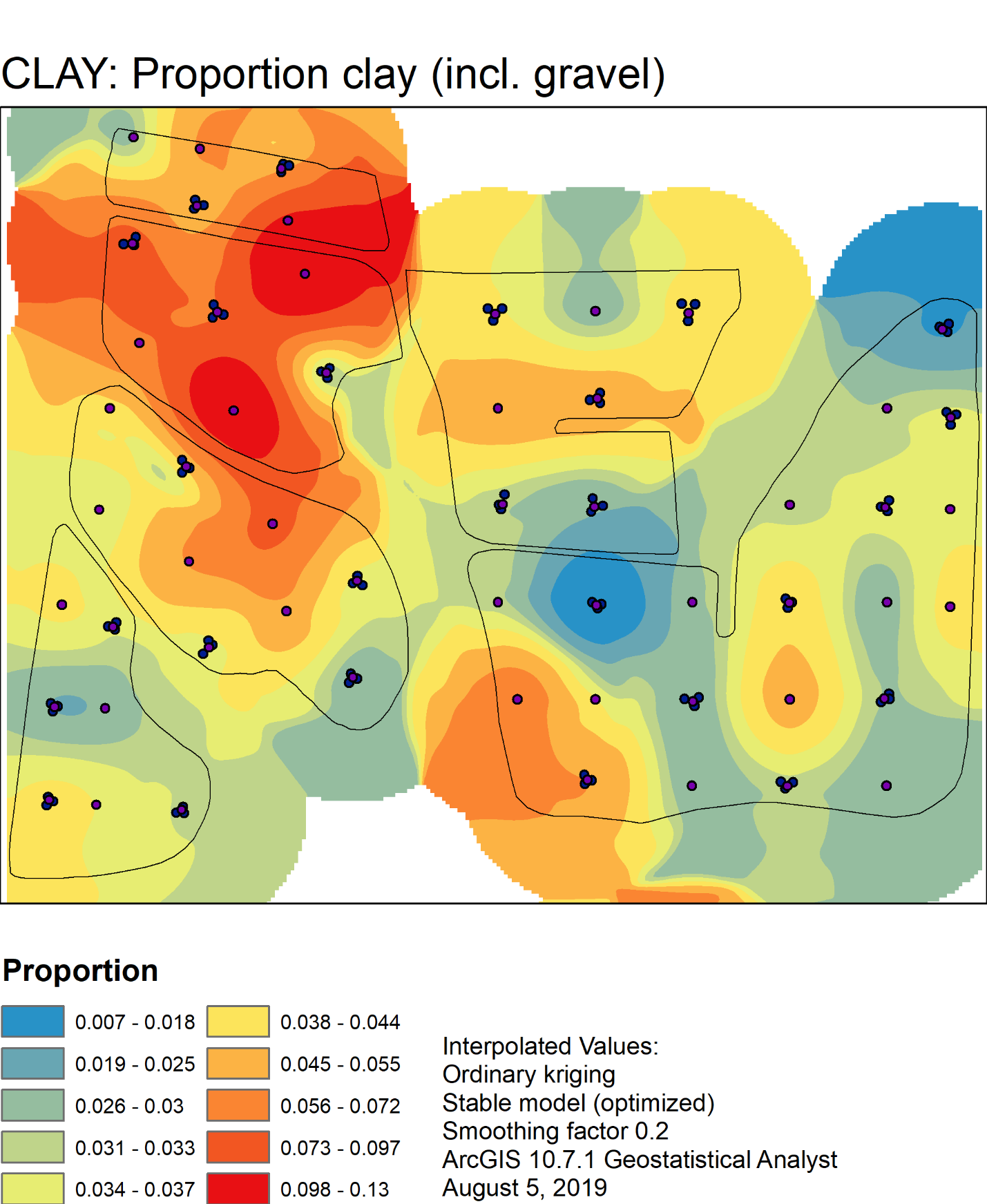


**S6G Fig. Map showing kriged surface of proportion of clay including gravel fraction (CLAY).**
