# Supplementary material for: Responses to environmental variability by herbivorous insects and their natural enemies within a bioenergy crop, Miscanthus x giganteus
Source: PLoS One. 2021 Feb 16;16(2):e0246855. doi: 10.1371/journal.pone.0246855 (PMC7886118; doi:10.1371/journal.pone.0246855)
Supplement: S7 Fig — SiteID refers to the main sample ID, shown in Fig 1. Rep (1, 2, or 3), refers to the three sample points located around each main point. Colors indicate the value of credence, or the ratio of the coefficient and standard error. No color indicates no or little credence. Light color indicates moderate levels of credence. Bold color indicates high levels of credence. (PDF) [file pone.0246855.s007.pdf]

SiteID 4

Season	Rep	INTRCPT	ELEV	SLOPE	SI	SILT	NDVI	NE	SPI
Early	1	-18.22	0.18	0.22	0.26	8.98	-0.25	-0.06	-0.05
		-18.24	0.18	0.22	0.26	8.98	-0.25	-0.06	-0.04
	2	-17.97	0.17	0.22	0.26	8.97	-0.28	-0.06	-0.05
		-18.00	0.17	0.22	0.26	8.98	-0.27	-0.06	-0.05
	3	-18.19	0.18	0.22	0.26	9.02	-0.24	-0.06	-0.04
		-18.21	0.18	0.22	0.26	9.02	-0.24	-0.06	-0.04
Mid	1	9.68	-0.07	0.05	0.33	5.18	-3.03	0.02	-0.03
		9.57	-0.07	0.05	0.34	5.18	-3.03	0.02	-0.03
	2	10.75	-0.08	0.04	0.32	5.12	-3.08	0.02	-0.03
		10.64	-0.08	0.04	0.32	5.12	-3.07	0.02	-0.03
	3	10.11	-0.07	0.04	0.33	5.20	-3.08	0.02	-0.03
		9.99	-0.07	0.04	0.33	5.20	-3.07	0.02	-0.03
Late	1	14.79	-0.05	-0.35	-0.74	-11.89	-1.26	-0.13	-0.07
		14.89	-0.05	-0.35	-0.74	-11.89	-1.26	-0.13	-0.07
	2	15.59	-0.06	-0.35	-0.75	-11.85	-1.27	-0.13	-0.07
		15.70	-0.06	-0.35	-0.75	-11.86	-1.27	-0.13	-0.07
	3	13.51	-0.04	-0.33	-0.72	-11.30	-1.25	-0.13	-0.07
		13.63	-0.05	-0.33	-0.72	-11.31	-1.25	-0.13	-0.07

SiteID 6

Season	Rep	INTRCPT	ELEV	SLOPE	SI	SILT	NDVI	NE	SPI
Early	1	-18.46	0.18	0.21	0.24	8.61	-0.32	-0.06	-0.05
		-18.49	0.18	0.21	0.24	8.61	-0.31	-0.06	-0.05
	2	-18.34	0.18	0.21	0.24	8.56	-0.35	-0.06	-0.05
		-18.37	0.18	0.21	0.24	8.56	-0.35	-0.06	-0.05
	3	-18.24	0.18	0.21	0.25	8.62	-0.33	-0.06	-0.05
		-18.27	0.18	0.21	0.25	8.62	-0.33	-0.06	-0.05
Mid	1	7.21	-0.05	0.08	0.35	4.44	-2.54	0.02	-0.03
		7.12	-0.05	0.08	0.35	4.43	-2.53	0.02	-0.03
	2	7.88	-0.06	0.08	0.34	4.17	-2.47	0.02	-0.03
		7.79	-0.05	0.08	0.34	4.16	-2.46	0.02	-0.03
	3	8.25	-0.06	0.07	0.34	4.36	-2.58	0.02	-0.03
		8.16	-0.06	0.07	0.34	4.35	-2.57	0.02	-0.03
Late	1	14.28	-0.04	-0.38	-0.80	-13.19	-1.23	-0.13	-0.07
		14.35	-0.04	-0.38	-0.80	-13.17	-1.24	-0.13	-0.07
	2	14.55	-0.05	-0.39	-0.82	-13.25	-1.26	-0.13	-0.07
		14.62	-0.05	-0.39	-0.82	-13.23	-1.26	-0.13	-0.07
	3	14.82	-0.05	-0.38	-0.80	-13.03	-1.27	-0.13	-0.07
		14.90	-0.05	-0.38	-0.80	-13.02	-1.27	-0.13	-0.07

SiteID 33

Season	Rep	INTRCPT	ELEV	SLOPE	SI	SILT	NDVI	NE	SPI
Early	1	2.83	0.02	0.09	-0.12	-5.50	-0.38	0.07	0.00
		2.88	0.02	0.09	-0.12	-5.46	-0.38	0.07	0.00
	2	3.11	0.02	0.08	-0.12	-5.42	-0.38	0.07	0.00
		3.17	0.02	0.08	-0.11	-5.38	-0.38	0.07	0.00
	3	2.54	0.02	0.09	-0.13	-6.24	-0.37	0.07	0.00
		2.58	0.02	0.09	-0.13	-6.19	-0.37	0.07	0.00
Mid	1	22.95	-0.13	-0.03	-0.43	-16.87	-3.16	0.00	0.00
		22.75	-0.13	-0.03	-0.43	-16.70	-3.13	0.00	0.00
	2	23.15	-0.14	-0.03	-0.43	-16.63	-3.15	0.00	0.00
		22.95	-0.13	-0.03	-0.43	-16.46	-3.12	0.00	0.00
	3	24.24	-0.14	-0.04	-0.44	-18.33	-3.36	-0.01	-0.01
		24.03	-0.14	-0.03	-0.44	-18.14	-3.34	-0.01	-0.01
Late	1	-18.64	0.17	0.24	-0.09	8.11	0.64	-0.05	-0.05
		-18.63	0.17	0.24	-0.10	8.13	0.64	-0.05	-0.05
	2	-18.77	0.17	0.24	-0.09	8.13	0.69	-0.05	-0.05
		-18.77	0.17	0.24	-0.09	8.15	0.69	-0.05	-0.05
	3	-18.92	0.17	0.24	-0.10	8.71	0.69	-0.06	-0.05
		-18.92	0.17	0.24	-0.10	8.74	0.68	-0.06	-0.05

SiteID 34

Season	Rep	INTRCPT	ELEV	SLOPE	SI	SILT	NDVI	NE	SPI
Early	1	4.41	0.02	0.06	0.00	-10.79	-0.15	0.06	-0.02
		4.51	0.02	0.06	0.00	-10.78	-0.14	0.06	-0.02
	2	4.06	0.02	0.06	-0.01	-10.53	-0.20	0.06	-0.01
		4.15	0.02	0.06	-0.01	-10.52	-0.19	0.06	-0.01
	3	5.42	0.01	0.06	0.00	-10.34	-0.14	0.07	-0.02
		5.52	0.01	0.06	0.00	-10.32	-0.14	0.07	-0.02
Mid	1	37.68	-0.23	-0.11	-0.49	-28.12	-4.47	0.01	-0.01
		37.67	-0.23	-0.11	-0.49	-27.96	-4.46	0.01	-0.01
	2	37.66	-0.23	-0.11	-0.49	-28.15	-4.51	0.01	-0.01
		37.59	-0.23	-0.11	-0.49	-27.96	-4.50	0.01	-0.01
	3	37.27	-0.23	-0.11	-0.47	-26.55	-4.38	0.00	-0.01
		37.20	-0.23	-0.11	-0.47	-26.34	-4.36	0.00	-0.01
Late	1	-7.59	0.06	0.21	-0.03	3.79	2.60	0.02	-0.03
		-7.59	0.06	0.21	-0.03	3.79	2.59	0.02	-0.03
	2	-8.31	0.07	0.21	-0.03	4.12	2.41	0.01	-0.04
		-8.32	0.07	0.21	-0.03	4.13	2.41	0.01	-0.04
	3	-8.28	0.07	0.21	-0.02	4.06	2.44	0.02	-0.03
		-8.29	0.07	0.21	-0.02	4.06	2.44	0.02	-0.03

SiteID 47

Season	Rep	INTRCPT	ELEV	SLOPE	SI	SILT	NDVI	NE	SPI
Early	1	143.52	-1.50	-0.10	0.38	33.03	-2.89	-0.04	-0.04
		142.82	-1.50	-0.10	0.38	32.87	-2.89	-0.04	-0.04
	2	149.43	-1.57	-0.11	0.36	34.36	-2.91	-0.04	-0.04
		149.16	-1.56	-0.11	0.36	34.30	-2.91	-0.04	-0.04
	3	134.00	-1.40	-0.08	0.37	30.79	-2.83	-0.04	-0.04
		133.38	-1.39	-0.08	0.37	30.65	-2.82	-0.04	-0.04
Mid	1	-20.30	0.32	0.14	0.50	-21.74	-3.65	0.00	-0.02
		-19.73	0.31	0.14	0.49	-21.64	-3.65	0.00	-0.02
	2	-23.52	0.35	0.14	0.52	-22.26	-3.65	0.00	-0.02
		-23.13	0.35	0.14	0.52	-22.20	-3.65	0.00	-0.02
	3	-21.24	0.33	0.14	0.48	-21.90	-3.66	0.00	-0.02
		-20.63	0.32	0.14	0.48	-21.80	-3.66	0.00	-0.02
Late	1	-150.67	1.71	0.41	0.12	-60.16	1.14	0.03	-0.16
		-150.05	1.71	0.41	0.12	-60.02	1.13	0.03	-0.16
	2	-146.14	1.66	0.41	0.09	-59.12	1.09	0.03	-0.16
		-145.44	1.66	0.41	0.08	-58.96	1.09	0.03	-0.16
	3	-142.66	1.63	0.40	0.08	-58.35	1.15	0.03	-0.16
		-141.95	1.62	0.40	0.08	-58.19	1.14	0.03	-0.16

SiteID 49

Season	Rep	INTRCPT	ELEV	SLOPE	SI	SILT	NDVI	NE	SPI
Early	1	50.53	-0.50	0.06	0.30	10.88	-2.49	-0.02	-0.01
		49.64	-0.49	0.06	0.30	10.65	-2.48	-0.02	-0.01
	2	63.08	-0.64	0.04	0.37	14.10	-2.62	-0.03	-0.02
		61.82	-0.62	0.04	0.36	13.78	-2.61	-0.03	-0.02
	3	62.53	-0.63	0.04	0.38	14.01	-2.63	-0.03	-0.02
		61.41	-0.62	0.04	0.38	13.72	-2.62	-0.03	-0.02
Mid	1	2.73	0.07	0.11	0.32	-18.14	-3.49	0.01	-0.02
		2.82	0.07	0.11	0.32	-18.14	-3.48	0.01	-0.02
	2	1.96	0.08	0.11	0.35	-18.14	-3.52	0.01	-0.02
		2.06	0.08	0.11	0.34	-18.14	-3.51	0.01	-0.02
	3	2.10	0.08	0.11	0.33	-18.12	-3.52	0.01	-0.02
		2.21	0.08	0.11	0.33	-18.11	-3.52	0.01	-0.02
Late	1	-118.44	1.35	0.32	0.60	-48.94	0.75	0.00	-0.14
		-118.92	1.36	0.32	0.59	-49.09	0.75	0.00	-0.14
	2	-125.21	1.43	0.34	0.56	-51.02	0.81	0.00	-0.15
		-125.87	1.43	0.34	0.56	-51.22	0.82	0.00	-0.15
	3	-124.57	1.42	0.34	0.52	-50.84	0.82	0.00	-0.14
		-125.22	1.43	0.34	0.52	-51.04	0.82	0.00	-0.14

S7 Figure. Geographically weighted regression (GWR) coefficient values for select Site IDs (outlined by red boxes in Figure 1), for models of abundance of phloem feeders in early, mid- and late seasons. ELEV is elevation in meters above mean sea level; SLOPE is percent slope; SI is southward index; SILT is percent silt in top 0-5cm of soil; NDVI is normalized difference of vegetation index; NE is abundance of natural enemies; SPI is abundance of spiders.

Legend:

- Black fill indicates C:SE > 3
- Grey fill indicates C:SE between 2 and 3
- No fill indicates C:SE between -2 and 2
- Light red fill indicates C:SE between -2 and -3
- Red fill indicates C:SE <-3
